# Supplementary material for: Sirt6 ablation in the liver causes fatty liver that increases cancer risk by upregulating Serpina12
Source: EMBO Rep. 2024 Feb 8;25(3):24. doi: 10.1038/s44319-024-00071-3 (PMC10933290; doi:10.1038/s44319-024-00071-3)
Supplement: Supplementary file 1 — Appendix [file 44319_2024_71_MOESM1_ESM.pdf]

Appendix Table of Contents

Appendix Figure S1……………………………………………………………2

Appendix Figure S2……………………………………………………………3

Appendix Figure S3……………………………………………………………4

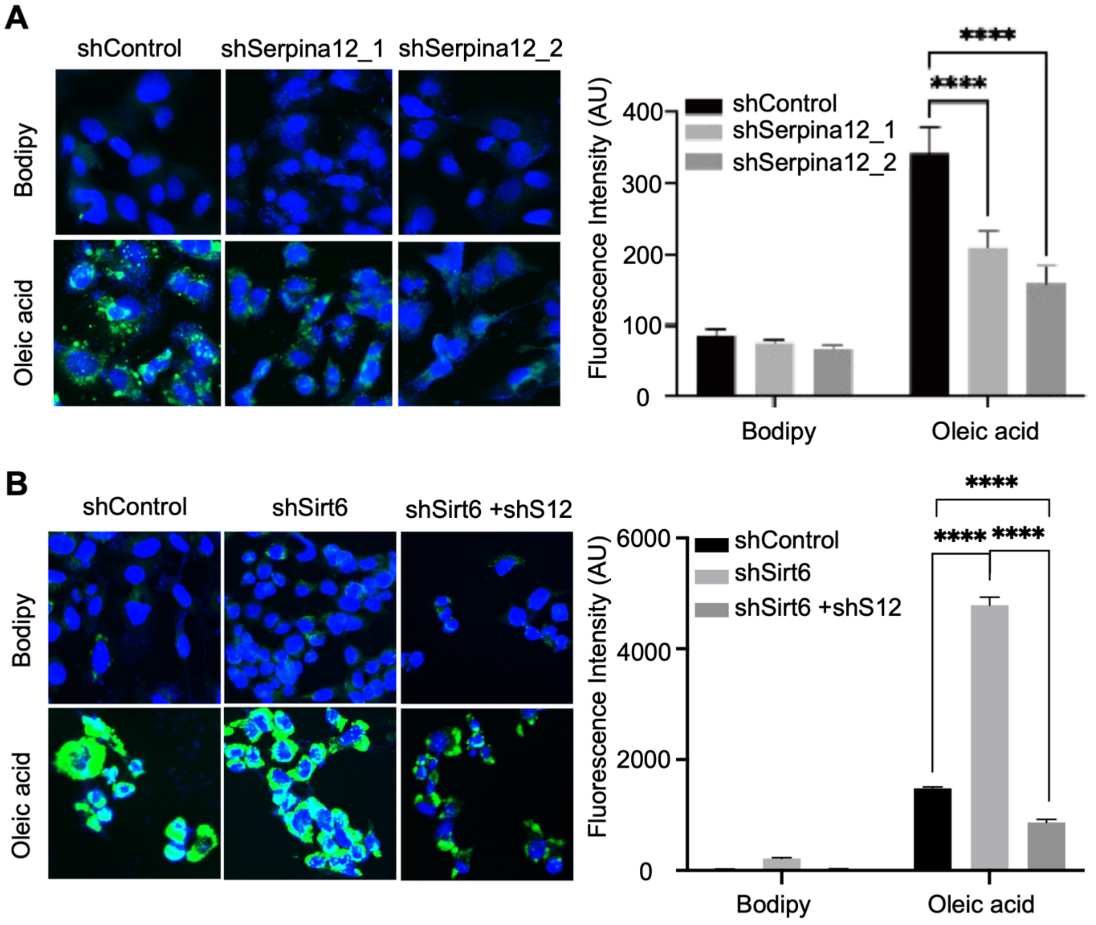

Appendix Figure S1 - Serpina12 promotes lipogenesis in Sirt6 deficient HepG2 cells.

(A) Lipogenesis induced by oleic acid in HepG2 cells. Cells were treated with 0.2mM oleic acid 12h followed by staining with fluorescent dye BODIPY 493/503; Relative intensity in control and knockdown Serpina12 HepG2 cells. n=10 TR; error bars = SEM. Two-way ANOVA. ****p<0.001.

(B) Lipogenesis induced by oleic acid in HepG2 cells with either knockdown Sirt6 or double knockdown Sirt6 and Serpina12. Cells were treated with 0.2mM oleic acid 12h followed by staining with fluorescent dye BODIPY 493/503; Relative intensity in control and knockdown Sirt6 or double knockdown Sirt6 and Serpina12 HepG2 cells. n=3 TR; error bars = SEM. Two-way ANOVA. ****p<0.001.

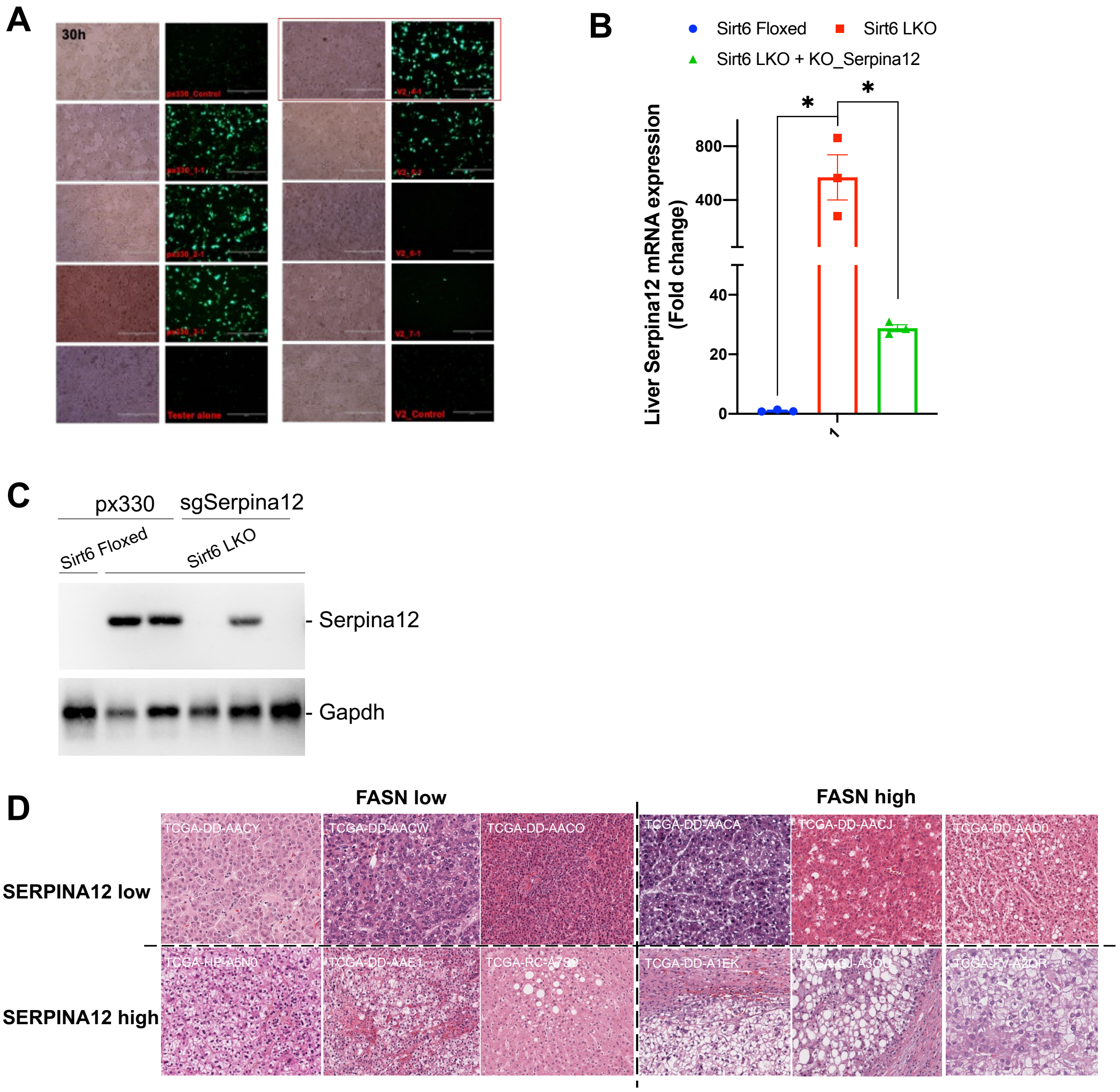

Appendix Figure S2 - Knockdown of Serpina12 alleviates Sirt6 deficient caused fatty liver in mice.
(A) The tester reporter pCAG-EGx-xFP-sgSerpina12 with NGG PAM sequence and pX330-sgSerpina12 were co-transfected into 293T cells, the cells were generated strong GFP signal suggesting the high cutting efficiency at 30 h.
(B) qPCR showed the relative Serpina12 mRNA levels. n=3 TR; error bars = SEM. Multiple *t*-test. *p<0.05.
(C) Western blot showed the Serpina12 protein levels.
(D) H&E stained sections of human SERPINA12 low -expressing HCC (top) or SERPINA12 high -expressing HCC (bottom), with either FASN low (left)– or FASN high (right)–expressing HCC.

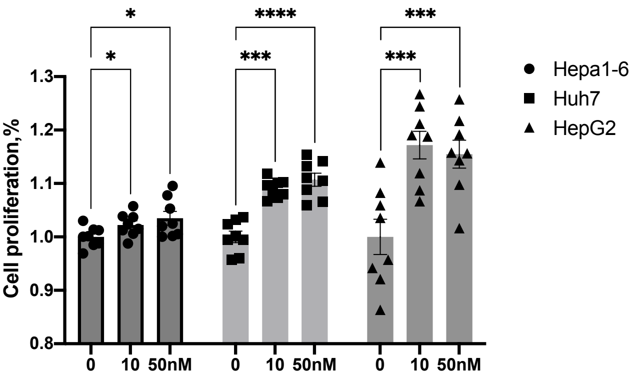

Appendix Figure S3 - Cell proliferation rate with different dose of insulin treatment. Hepa1-6, Huh7 and HepG2 cells were treated with (10nM or 50nM) or without insulin for 24 h. n=8 BR; error bars = SEM. Two-way ANOVA. *p<0.05, ***p<0.005,****p<0.001.
