# Supplementary figures and images for: Sirt6 ablation in the liver causes fatty liver that increases cancer risk by upregulating Serpina12
Source: EMBO Rep. 2024 Feb 8;25(3):24. doi: 10.1038/s44319-024-00071-3 (PMC10933290; doi:10.1038/s44319-024-00071-3)

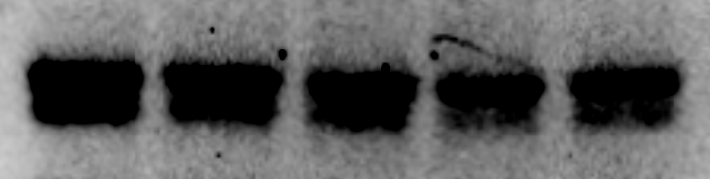

Supplement: Supplementary file 3 — Source Data Fig. 2 [file 44319_2024_71_MOESM3_ESM.zip › Figure 2/2H/Western actin.tif]

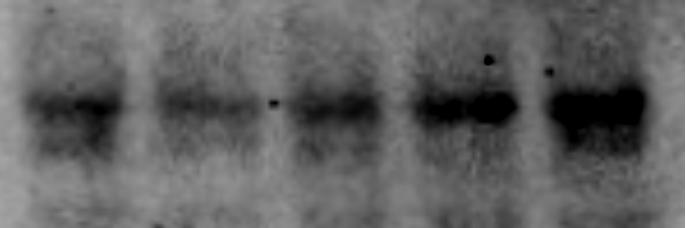

Supplement: Supplementary file 3 — Source Data Fig. 2 [file 44319_2024_71_MOESM3_ESM.zip › Figure 2/2H/Western SERPINA12.tif]

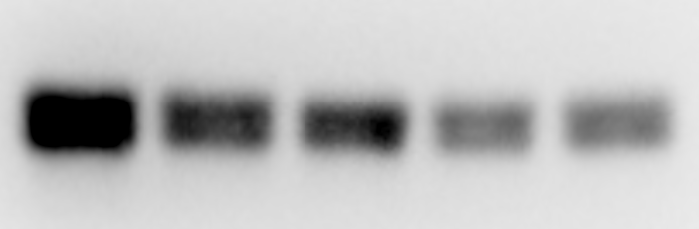

Supplement: Supplementary file 3 — Source Data Fig. 2 [file 44319_2024_71_MOESM3_ESM.zip › Figure 2/2H/Western SIRT6.tif]

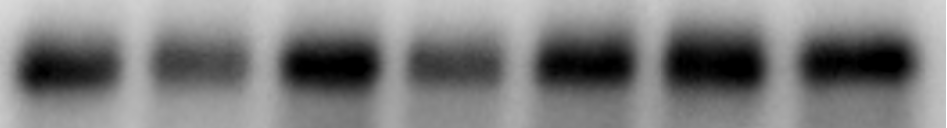

Supplement: Supplementary file 3 — Source Data Fig. 2 [file 44319_2024_71_MOESM3_ESM.zip › Figure 2/2F/Western actin.tif]

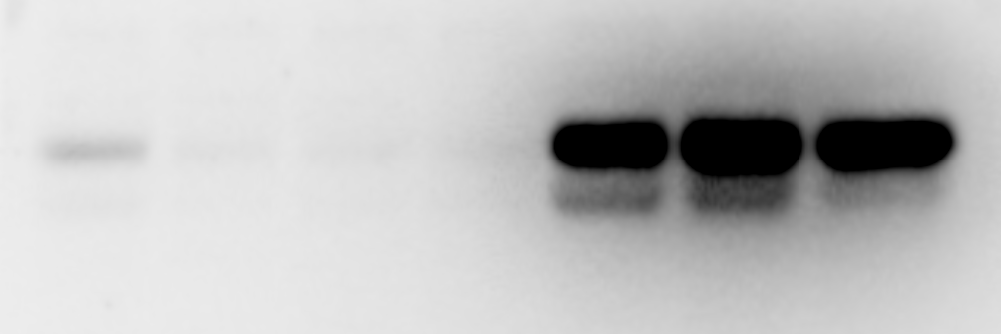

Supplement: Supplementary file 3 — Source Data Fig. 2 [file 44319_2024_71_MOESM3_ESM.zip › Figure 2/2F/Western Serpina12.tif]

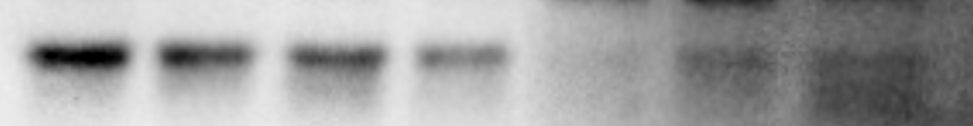

Supplement: Supplementary file 3 — Source Data Fig. 2 [file 44319_2024_71_MOESM3_ESM.zip › Figure 2/2F/Western Sirt6.tif]

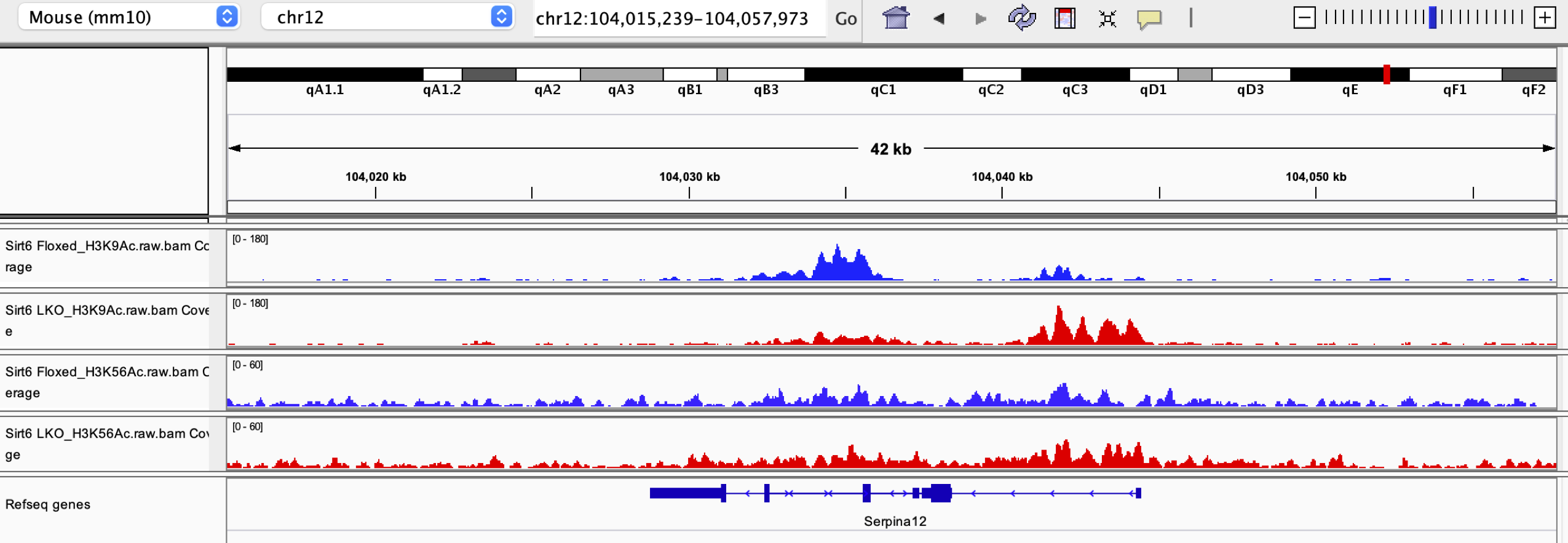

Supplement: Supplementary file 4 — Source Data Fig. 3 [file 44319_2024_71_MOESM4_ESM.zip › Figure 3/3A/ChIP seq data_Serpina12 locus.tiff]

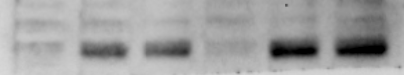

Supplement: Supplementary file 5 — Source Data Fig. 4 [file 44319_2024_71_MOESM5_ESM.zip › Figure 4/4B/Western pIRS1-Y612.tif]

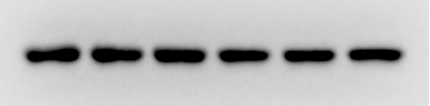

Supplement: Supplementary file 5 — Source Data Fig. 4 [file 44319_2024_71_MOESM5_ESM.zip › Figure 4/4B/Western actin.tif]

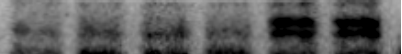

Supplement: Supplementary file 5 — Source Data Fig. 4 [file 44319_2024_71_MOESM5_ESM.zip › Figure 4/4B/Western p-AKT T308.tif]

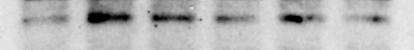

Supplement: Supplementary file 5 — Source Data Fig. 4 [file 44319_2024_71_MOESM5_ESM.zip › Figure 4/4B/Western p-AKT S473.tif]

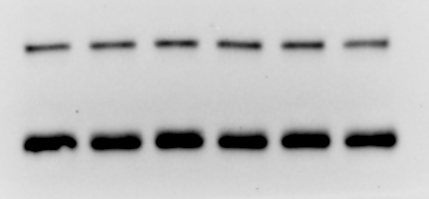

Supplement: Supplementary file 5 — Source Data Fig. 4 [file 44319_2024_71_MOESM5_ESM.zip › Figure 4/4B/Western IRb.tif]

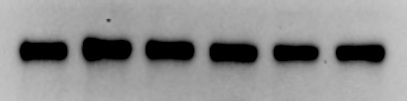

Supplement: Supplementary file 5 — Source Data Fig. 4 [file 44319_2024_71_MOESM5_ESM.zip › Figure 4/4B/Western IRS1.tif]

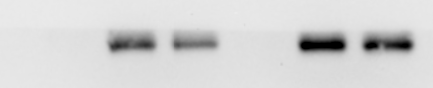

Supplement: Supplementary file 5 — Source Data Fig. 4 [file 44319_2024_71_MOESM5_ESM.zip › Figure 4/4B/Western p-IRb Y1150-1151.tif]

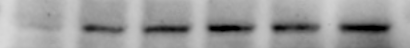

Supplement: Supplementary file 5 — Source Data Fig. 4 [file 44319_2024_71_MOESM5_ESM.zip › Figure 4/4B/Western SERPINA12.tif]

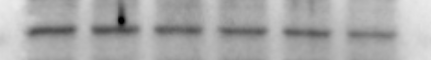

Supplement: Supplementary file 5 — Source Data Fig. 4 [file 44319_2024_71_MOESM5_ESM.zip › Figure 4/4B/Western T-AKT.tif]

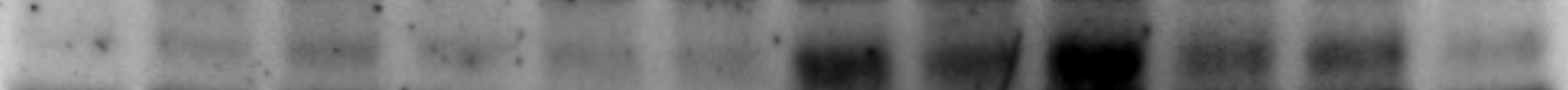

Supplement: Supplementary file 5 — Source Data Fig. 4 [file 44319_2024_71_MOESM5_ESM.zip › Figure 4/4C/Western p-AKT S473 .tif]

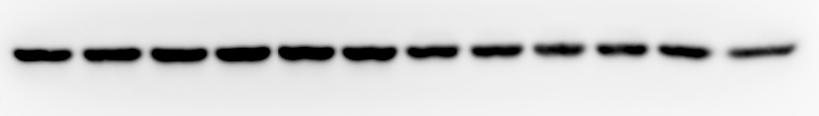

Supplement: Supplementary file 5 — Source Data Fig. 4 [file 44319_2024_71_MOESM5_ESM.zip › Figure 4/4C/Western actin.tif]

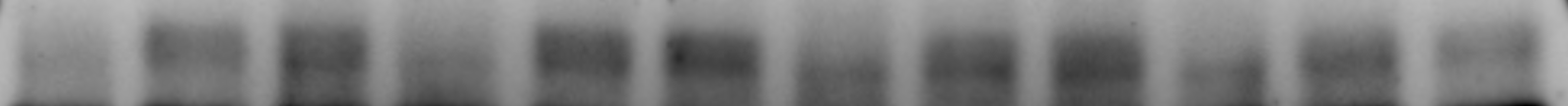

Supplement: Supplementary file 5 — Source Data Fig. 4 [file 44319_2024_71_MOESM5_ESM.zip › Figure 4/4C/Western p-AKT T308.tif]

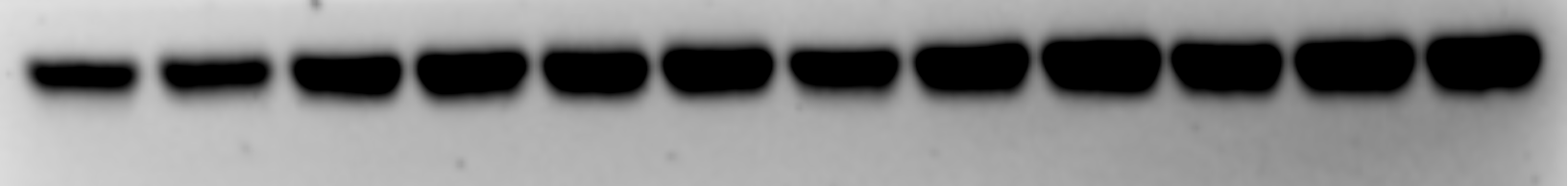

Supplement: Supplementary file 5 — Source Data Fig. 4 [file 44319_2024_71_MOESM5_ESM.zip › Figure 4/4C/Western AKT.tif]

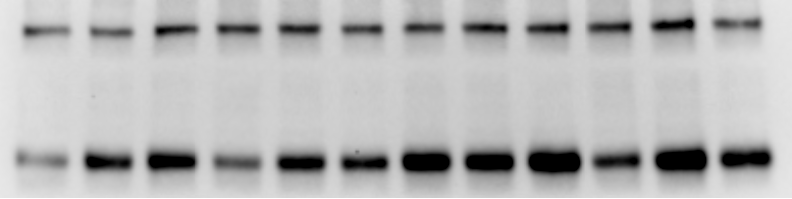

Supplement: Supplementary file 5 — Source Data Fig. 4 [file 44319_2024_71_MOESM5_ESM.zip › Figure 4/4C/Western IRb.tif]

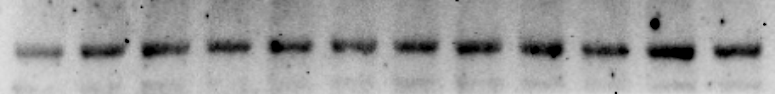

Supplement: Supplementary file 5 — Source Data Fig. 4 [file 44319_2024_71_MOESM5_ESM.zip › Figure 4/4C/Western IRS1.tif]

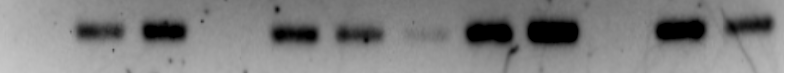

Supplement: Supplementary file 5 — Source Data Fig. 4 [file 44319_2024_71_MOESM5_ESM.zip › Figure 4/4C/Western p-IRb Y1146.tif]

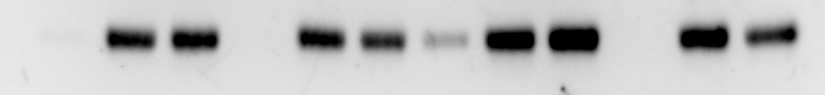

Supplement: Supplementary file 5 — Source Data Fig. 4 [file 44319_2024_71_MOESM5_ESM.zip › Figure 4/4C/Western p-IRb Y1150-1151.tif]

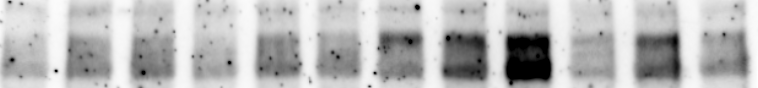

Supplement: Supplementary file 5 — Source Data Fig. 4 [file 44319_2024_71_MOESM5_ESM.zip › Figure 4/4C/Western p-IRS1 Y608.tif]

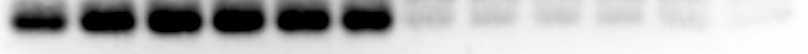

Supplement: Supplementary file 5 — Source Data Fig. 4 [file 44319_2024_71_MOESM5_ESM.zip › Figure 4/4C/Western Sirt6.tif]

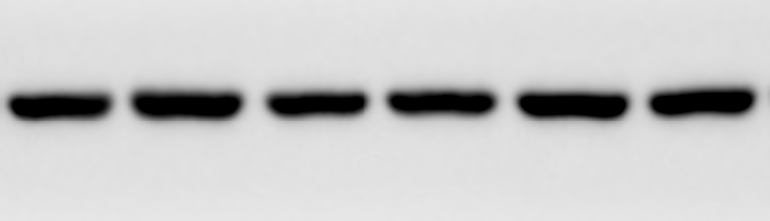

Supplement: Supplementary file 5 — Source Data Fig. 4 [file 44319_2024_71_MOESM5_ESM.zip › Figure 4/4D/Western actin.tif]

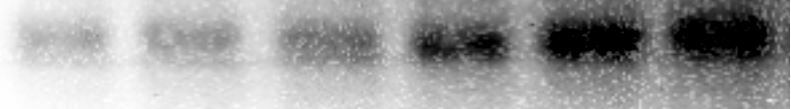

Supplement: Supplementary file 5 — Source Data Fig. 4 [file 44319_2024_71_MOESM5_ESM.zip › Figure 4/4D/Western Serpina12.tif]

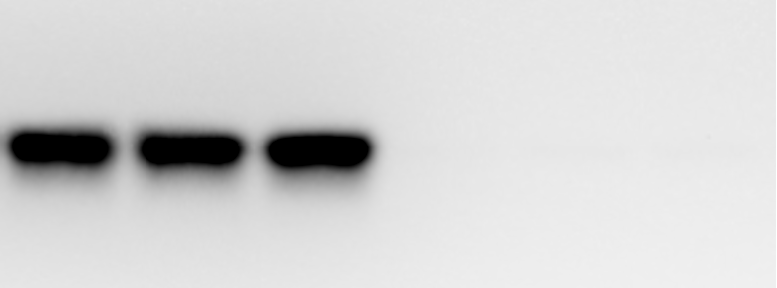

Supplement: Supplementary file 5 — Source Data Fig. 4 [file 44319_2024_71_MOESM5_ESM.zip › Figure 4/4D/Western Sirt6.tif]

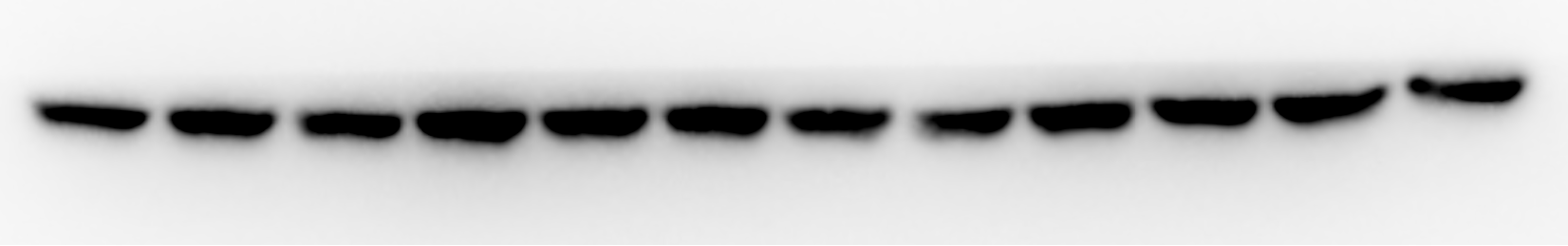

Supplement: Supplementary file 5 — Source Data Fig. 4 [file 44319_2024_71_MOESM5_ESM.zip › Figure 4/4A/Western actin.tif]

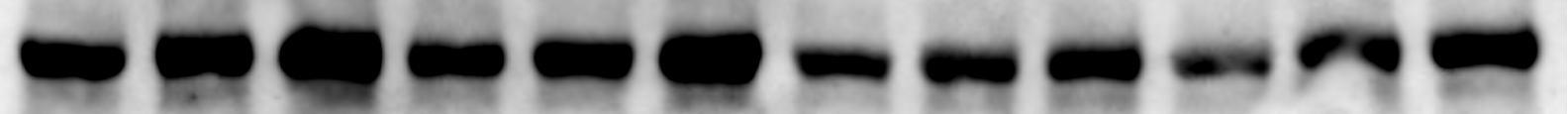

Supplement: Supplementary file 5 — Source Data Fig. 4 [file 44319_2024_71_MOESM5_ESM.zip › Figure 4/4A/Western IRS1.tif]

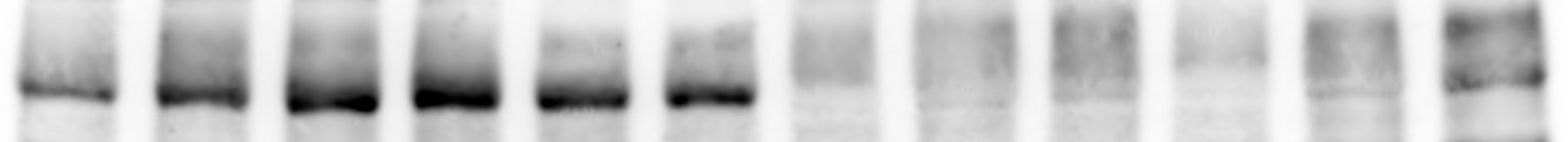

Supplement: Supplementary file 5 — Source Data Fig. 4 [file 44319_2024_71_MOESM5_ESM.zip › Figure 4/4A/Western p-IRS1 Y608.tif]

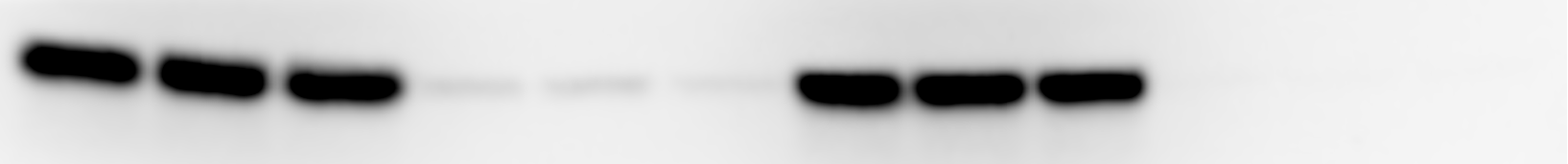

Supplement: Supplementary file 5 — Source Data Fig. 4 [file 44319_2024_71_MOESM5_ESM.zip › Figure 4/4A/Western Sirt6.tif]

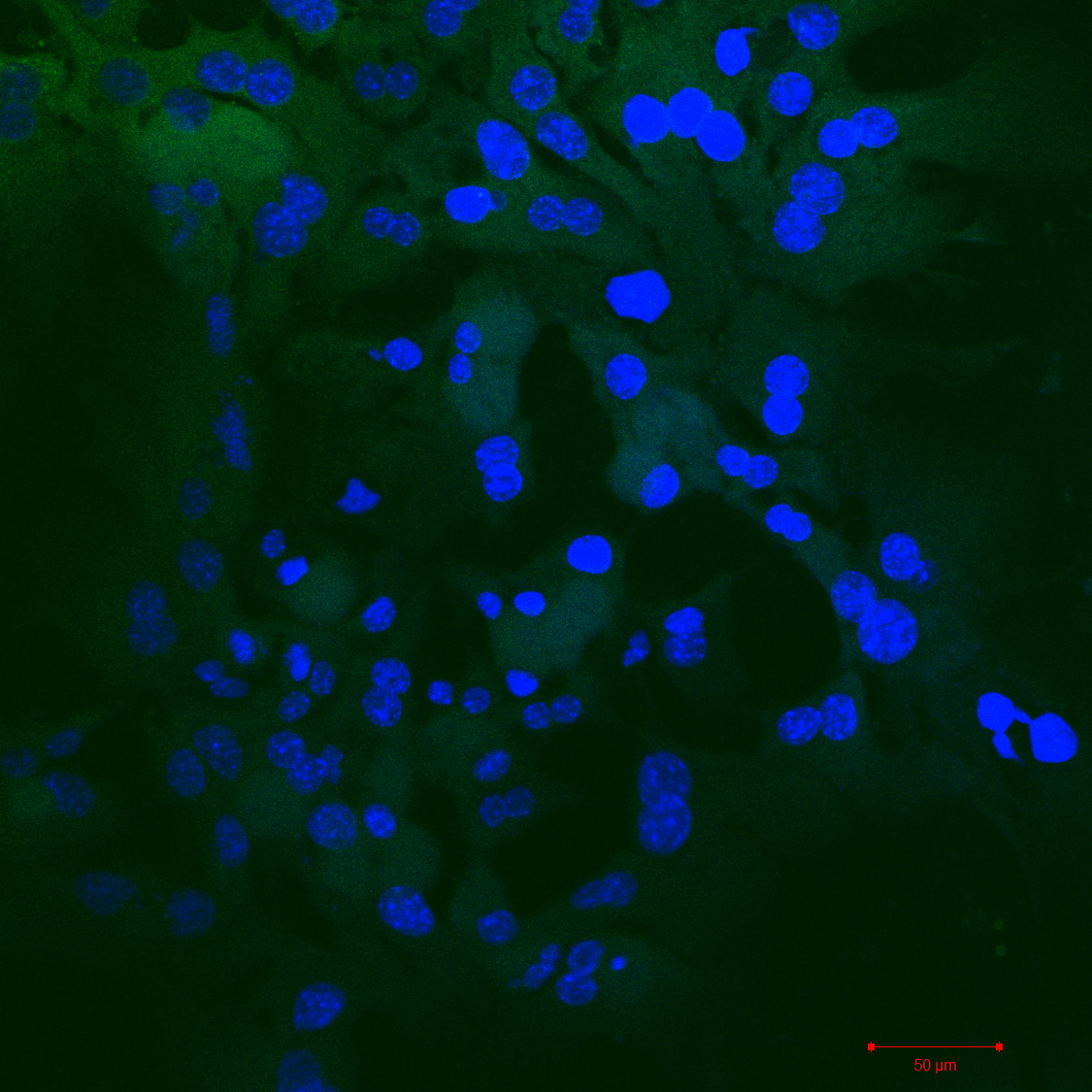

Supplement: Supplementary file 5 — Source Data Fig. 4 [file 44319_2024_71_MOESM5_ESM.zip › Figure 4/4E/BODIPY 493 503/Ad-Cre+ KD Serpina12 +4g:L.tif]

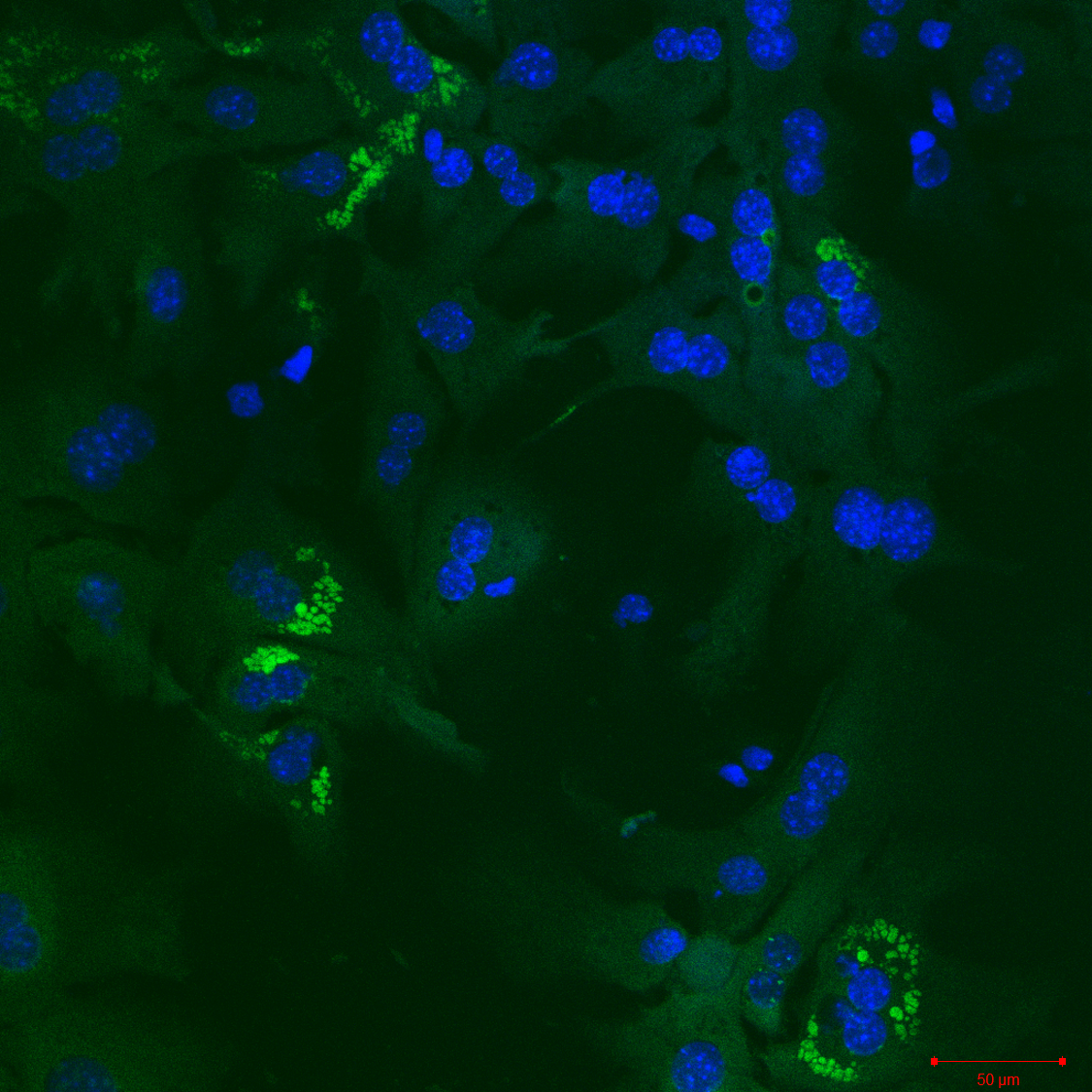

Supplement: Supplementary file 5 — Source Data Fig. 4 [file 44319_2024_71_MOESM5_ESM.zip › Figure 4/4E/BODIPY 493 503/Ad-Cre +1g:L.tif]

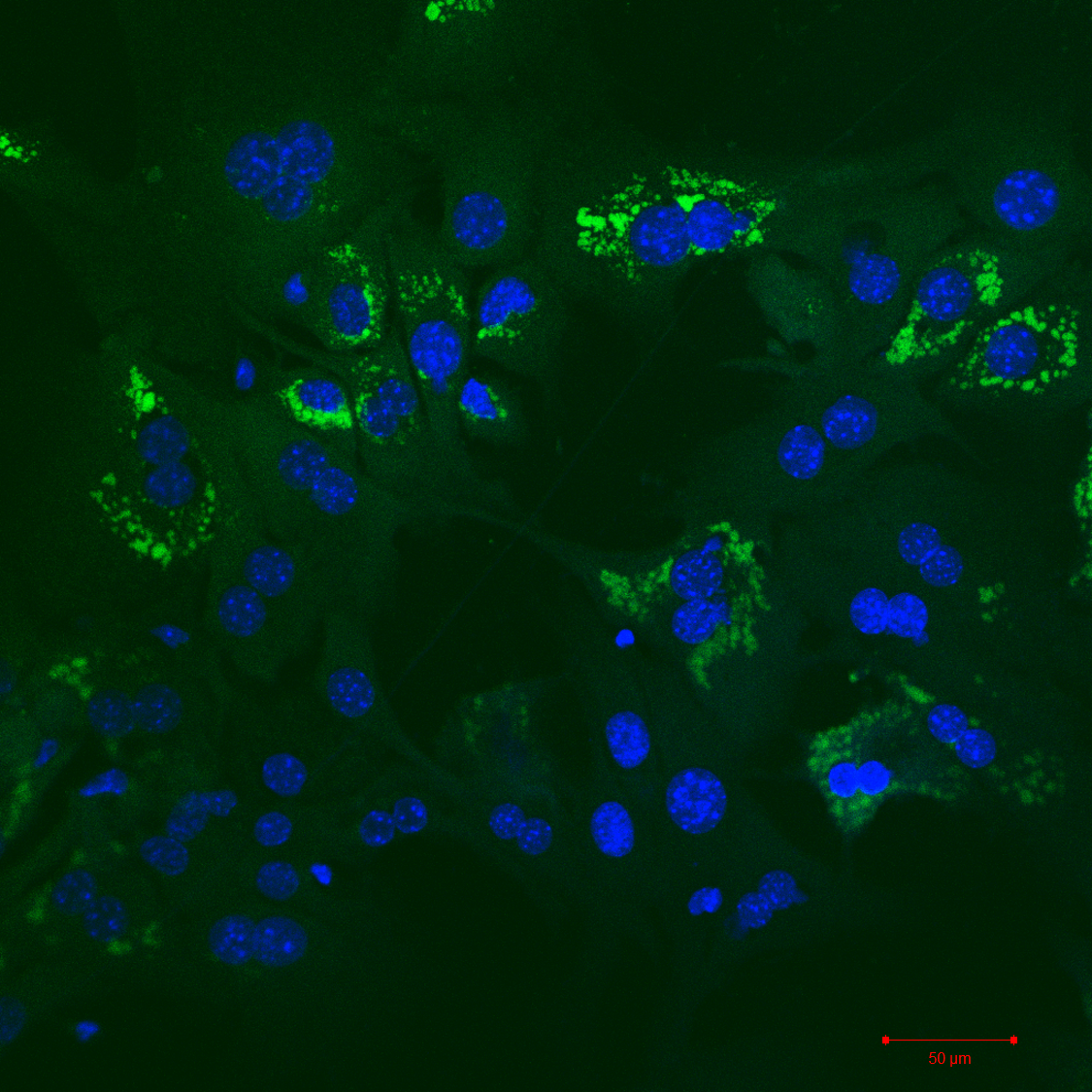

Supplement: Supplementary file 5 — Source Data Fig. 4 [file 44319_2024_71_MOESM5_ESM.zip › Figure 4/4E/BODIPY 493 503/Control +4g:L.tif]

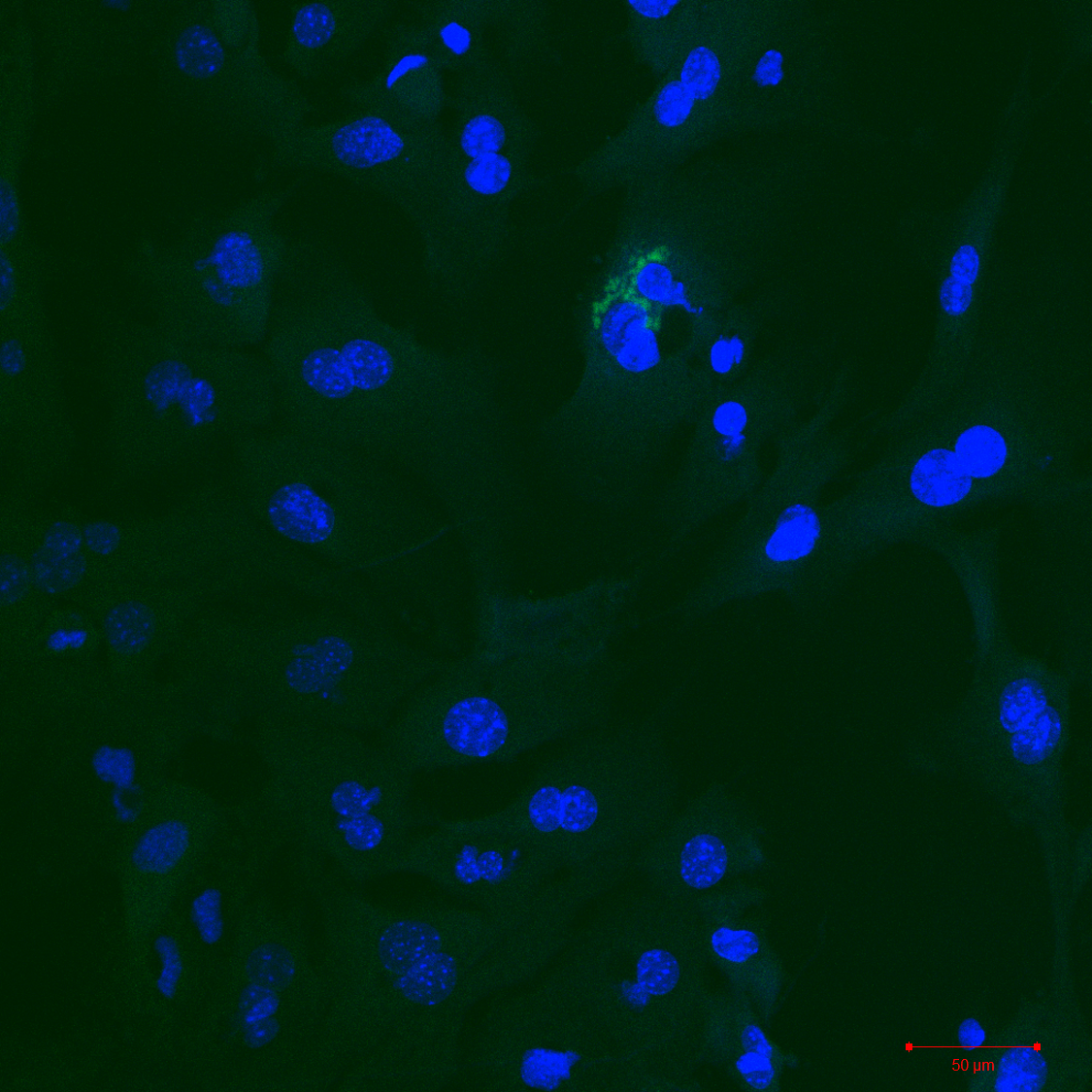

Supplement: Supplementary file 5 — Source Data Fig. 4 [file 44319_2024_71_MOESM5_ESM.zip › Figure 4/4E/BODIPY 493 503/Control +1g:L.tif]

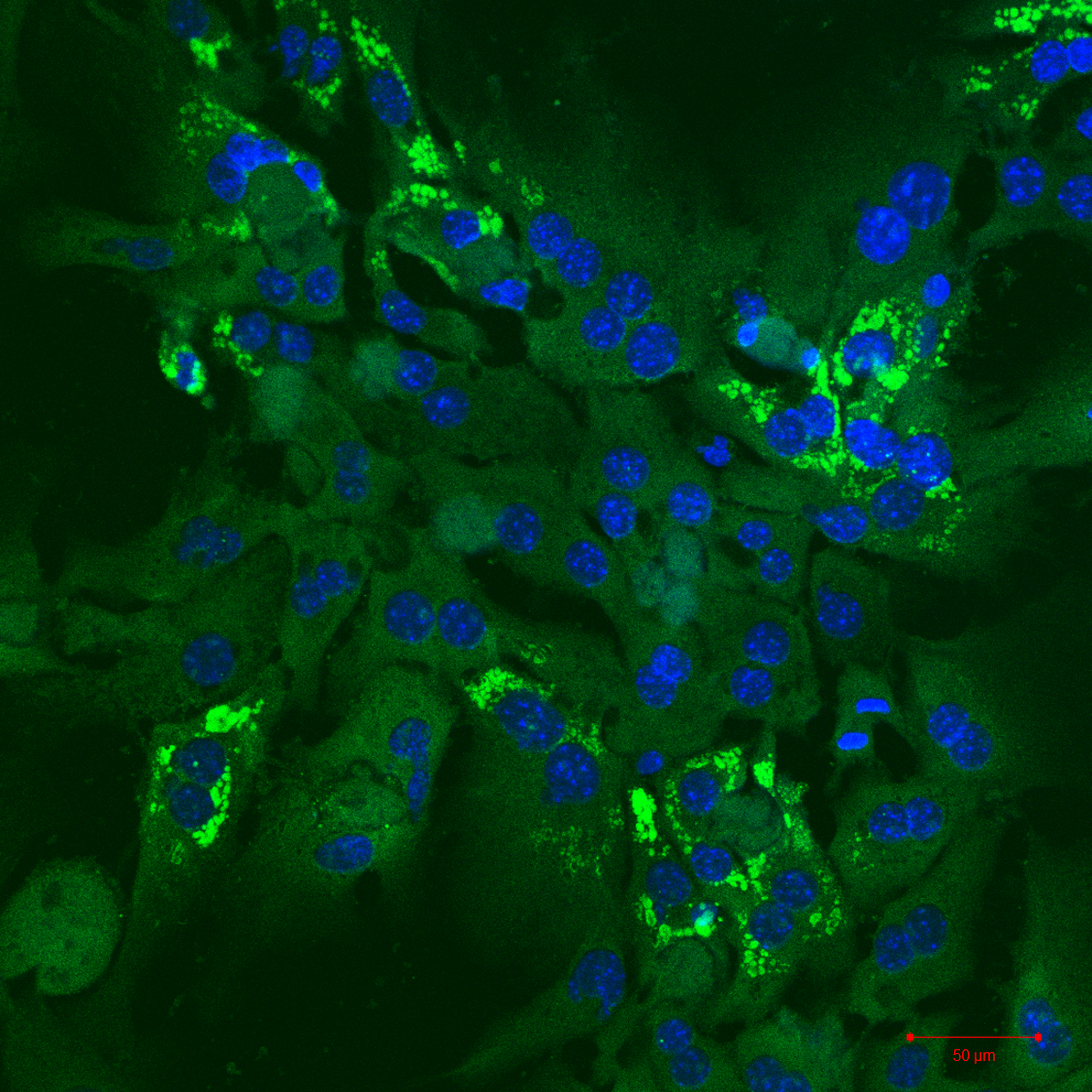

Supplement: Supplementary file 5 — Source Data Fig. 4 [file 44319_2024_71_MOESM5_ESM.zip › Figure 4/4E/BODIPY 493 503/Ad-Cre +4g:L.tif]

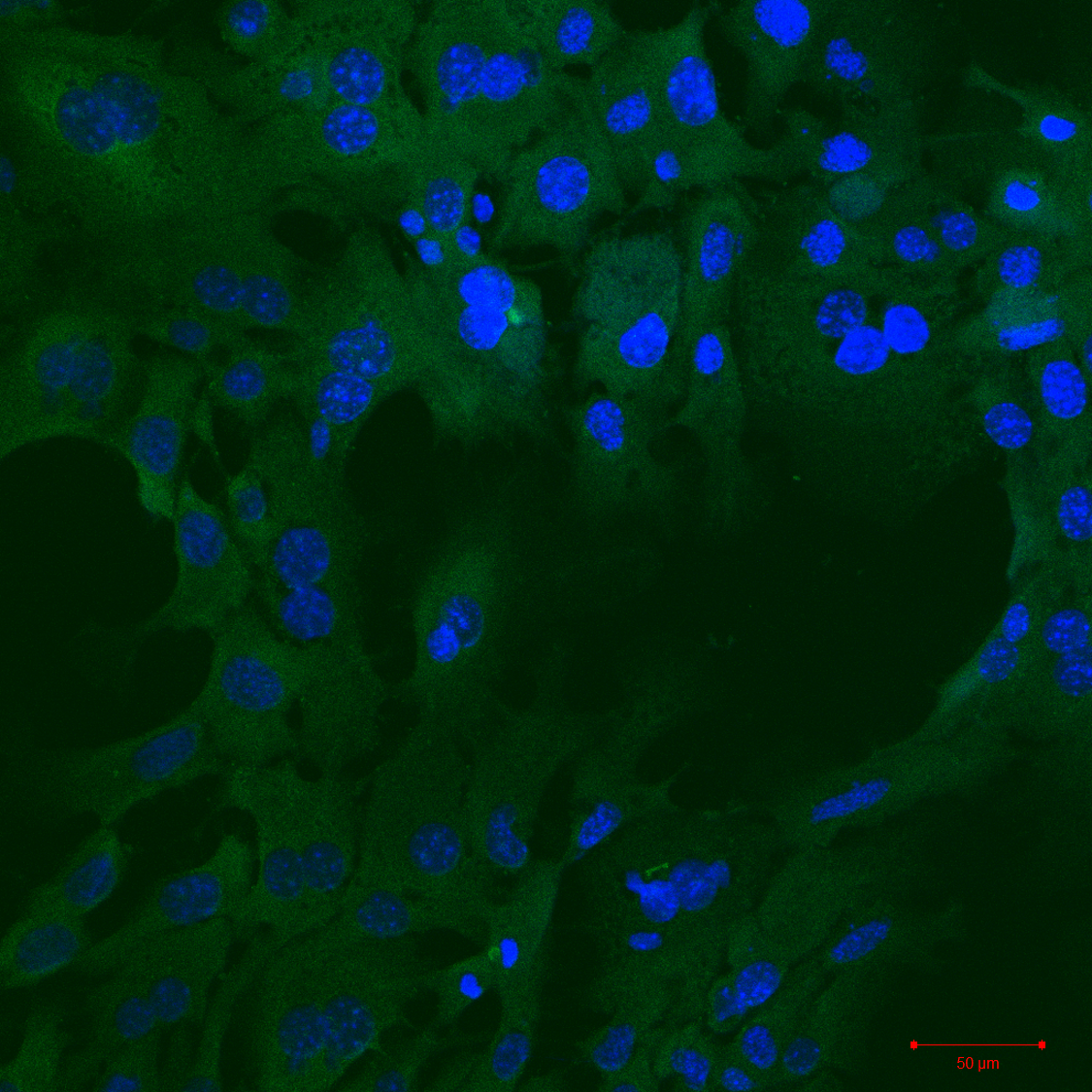

Supplement: Supplementary file 5 — Source Data Fig. 4 [file 44319_2024_71_MOESM5_ESM.zip › Figure 4/4E/BODIPY 493 503/Ad-Cre+ KD Serpina12 +1g:L.tif]

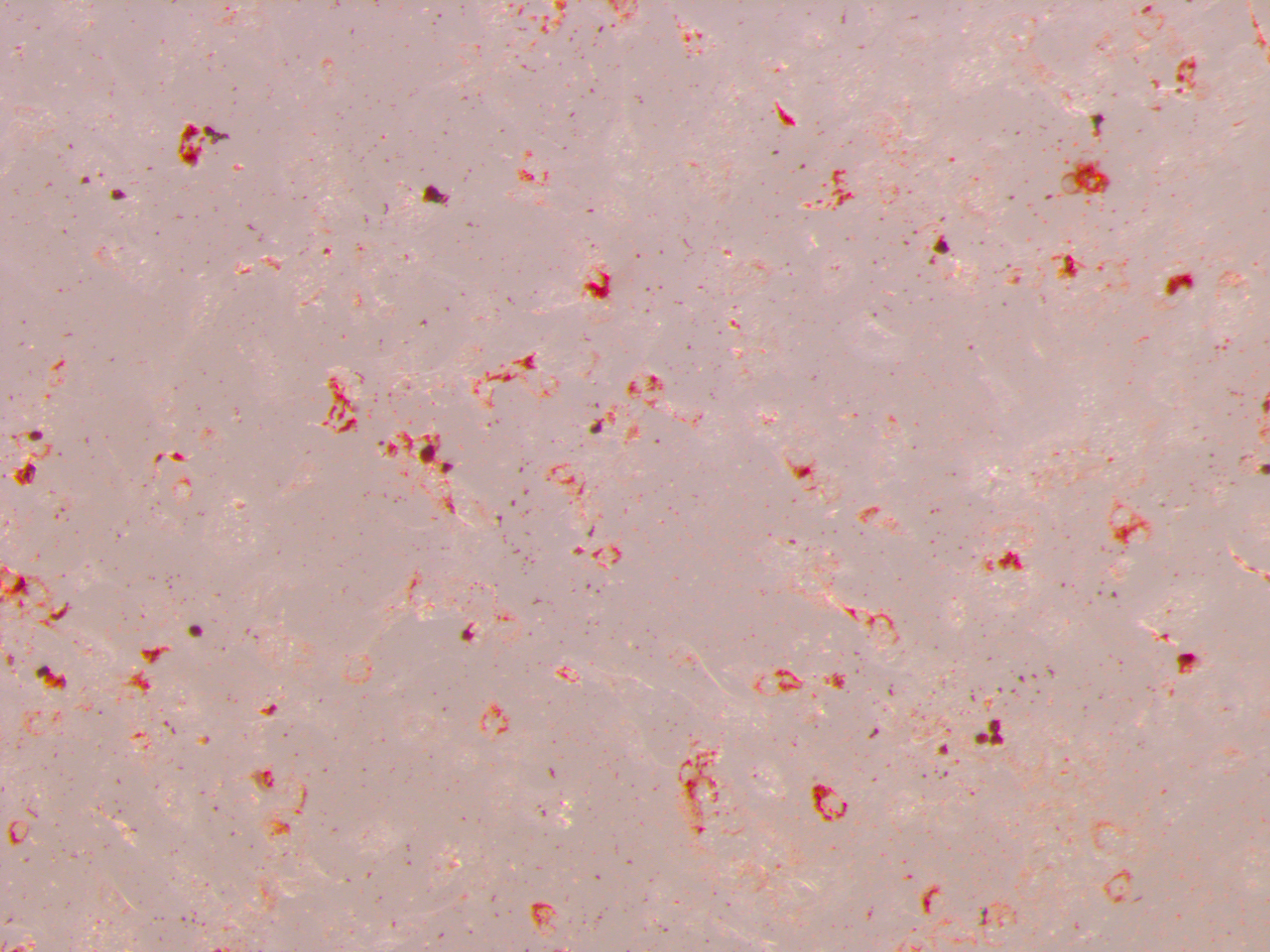

Supplement: Supplementary file 5 — Source Data Fig. 4 [file 44319_2024_71_MOESM5_ESM.zip › Figure 4/4E/Oil Red O/Ad-Cre+ KD Serpina12 +4g:L.tif]

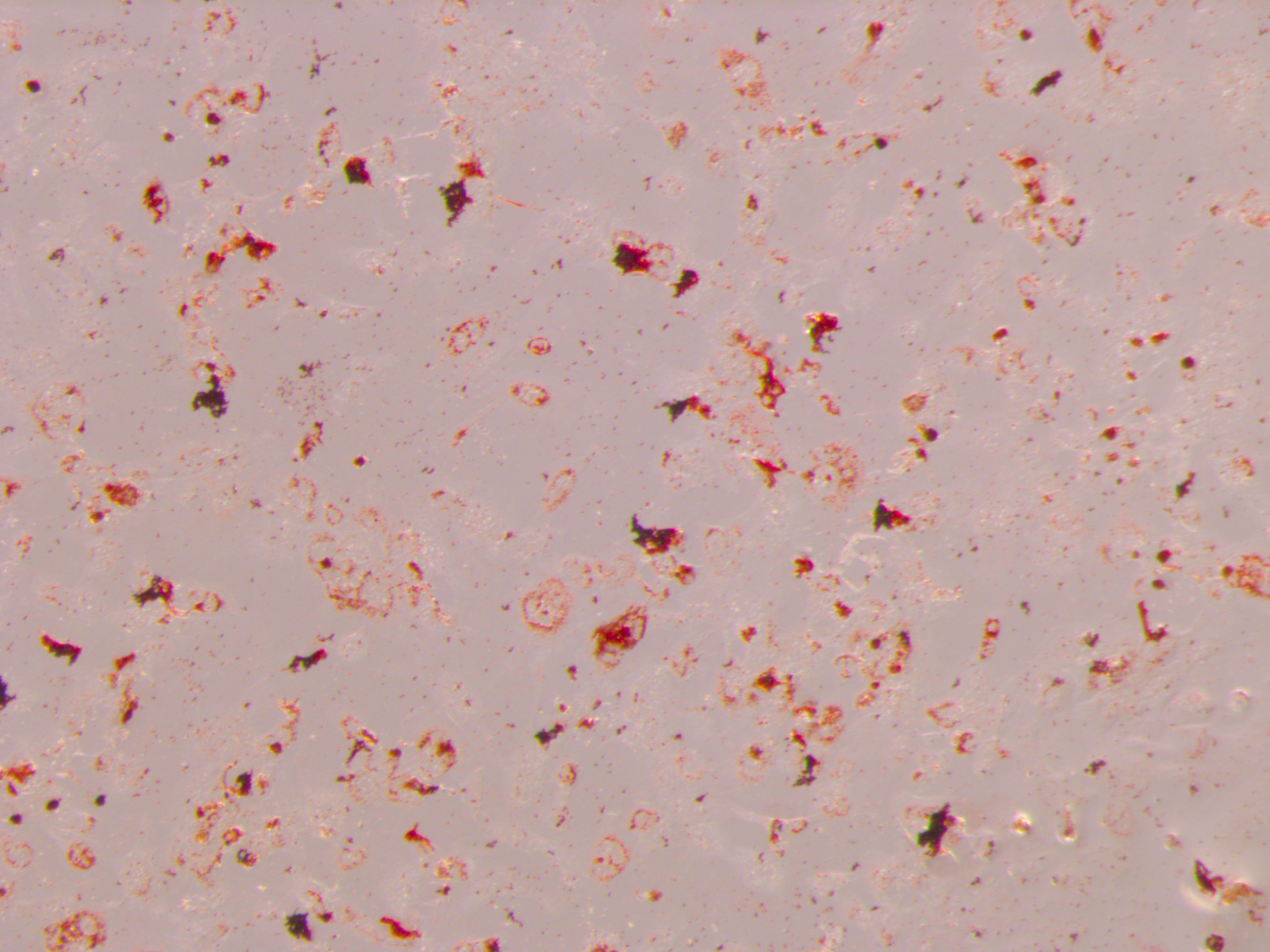

Supplement: Supplementary file 5 — Source Data Fig. 4 [file 44319_2024_71_MOESM5_ESM.zip › Figure 4/4E/Oil Red O/Control +4g:L.tif]

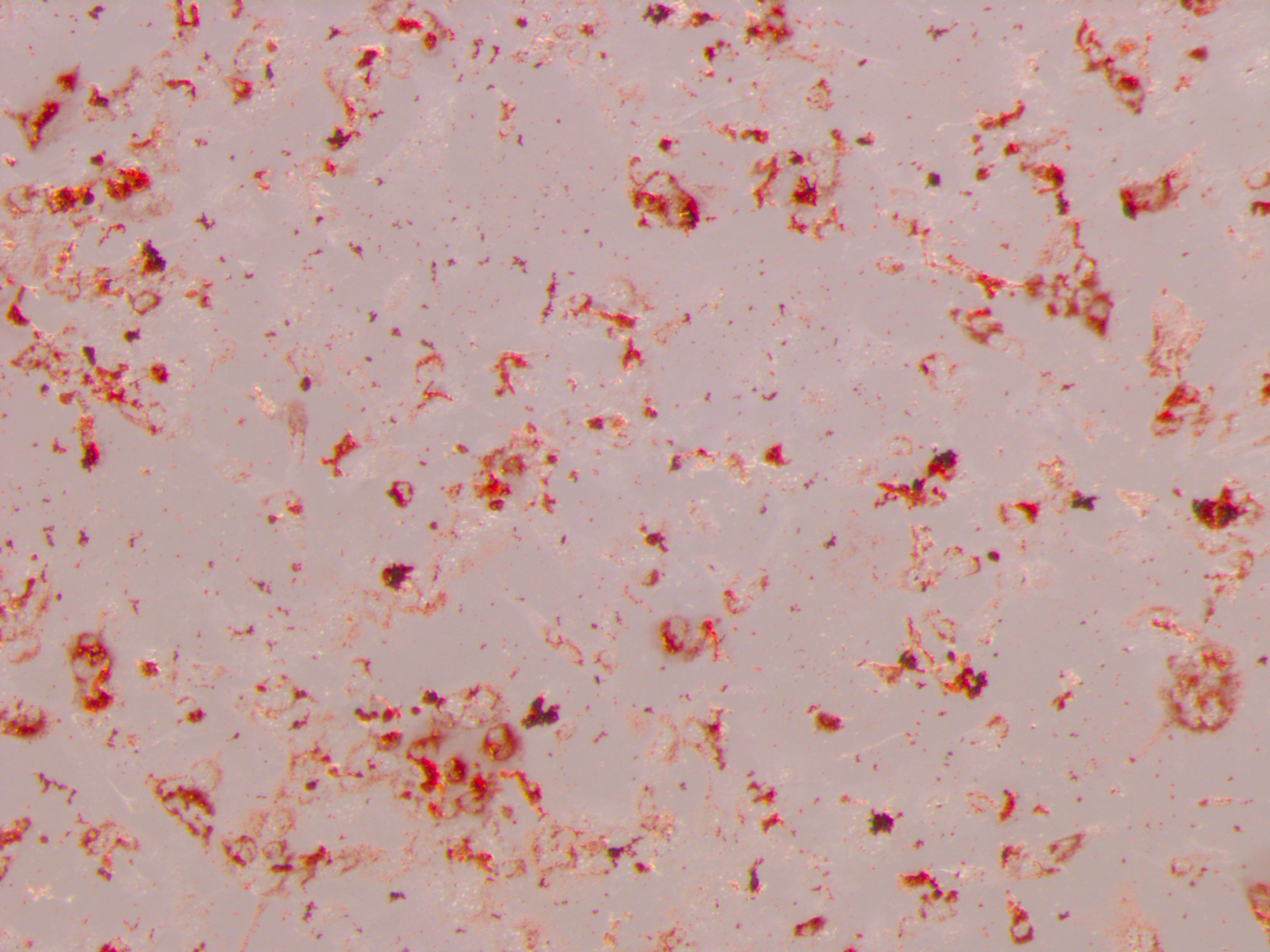

Supplement: Supplementary file 5 — Source Data Fig. 4 [file 44319_2024_71_MOESM5_ESM.zip › Figure 4/4E/Oil Red O/Ad-Cre +4g:L.tif]

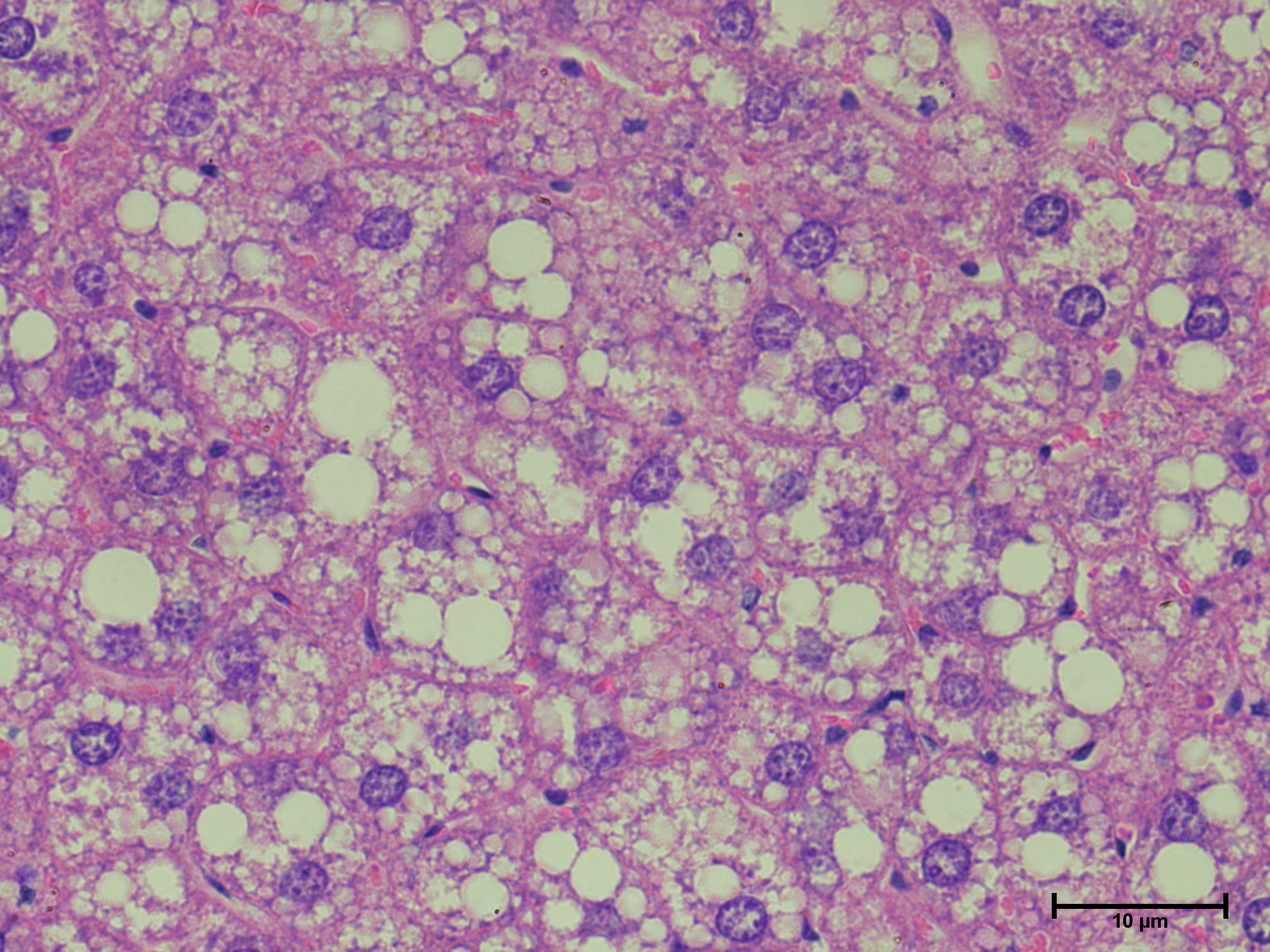

Supplement: Supplementary file 6 — Source Data Fig. 5 [file 44319_2024_71_MOESM6_ESM.zip › Figure 5/5B/HE Sirt6 LKO.tif]

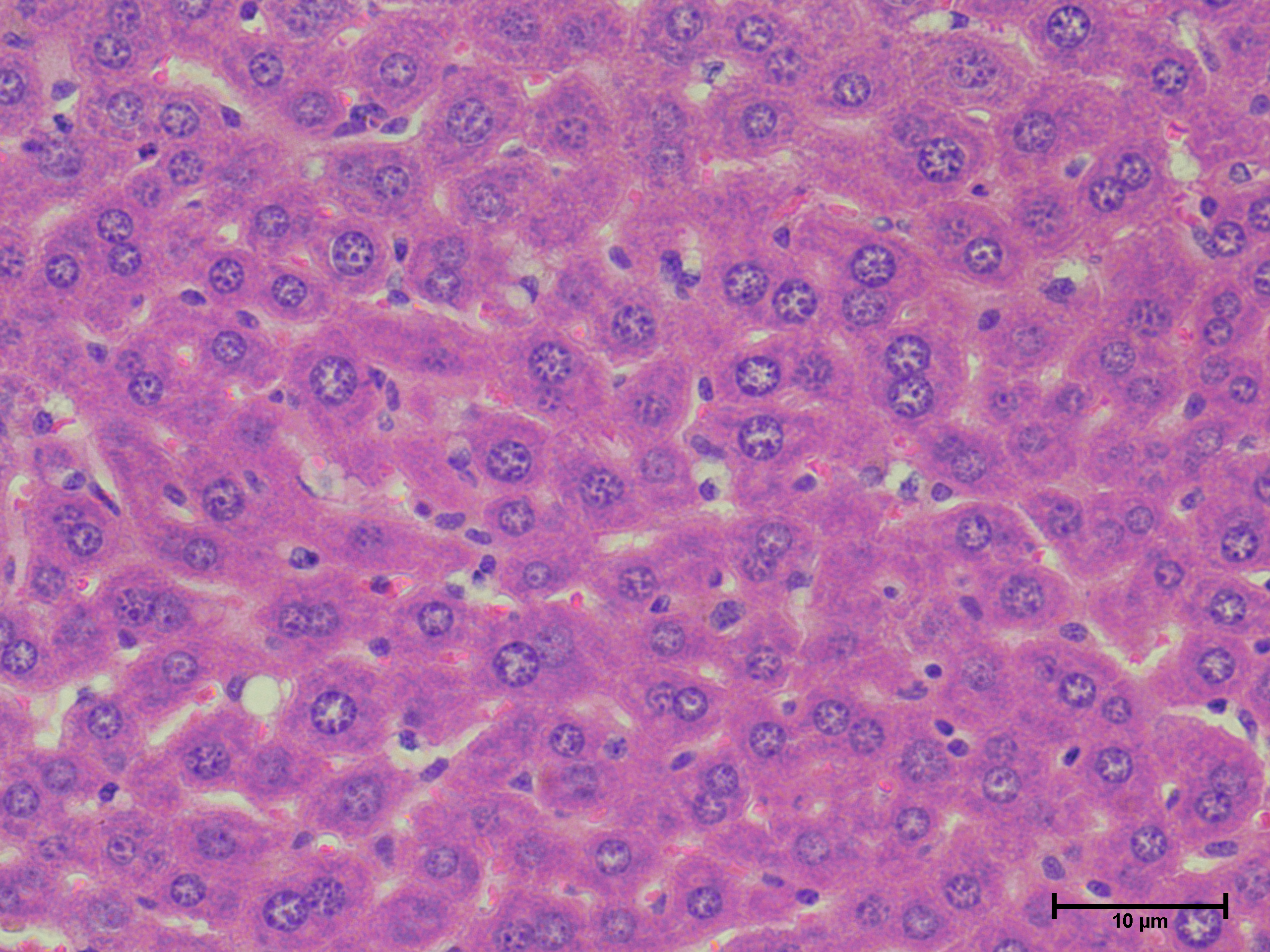

Supplement: Supplementary file 6 — Source Data Fig. 5 [file 44319_2024_71_MOESM6_ESM.zip › Figure 5/5B/HE Sirt6 Floxed.tif]

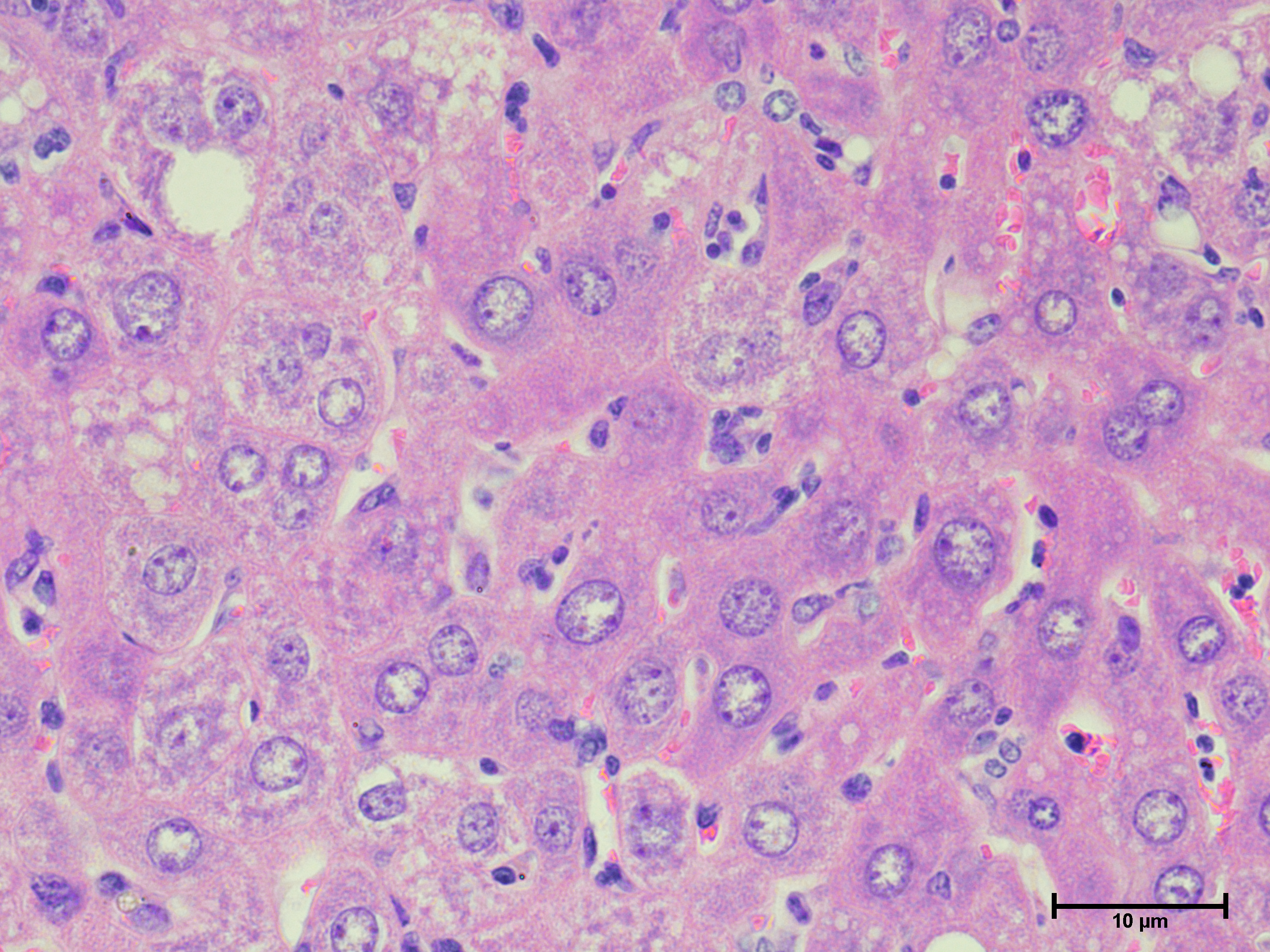

Supplement: Supplementary file 6 — Source Data Fig. 5 [file 44319_2024_71_MOESM6_ESM.zip › Figure 5/5B/HE Sirt6 LKO + sgSerpina12.tif]

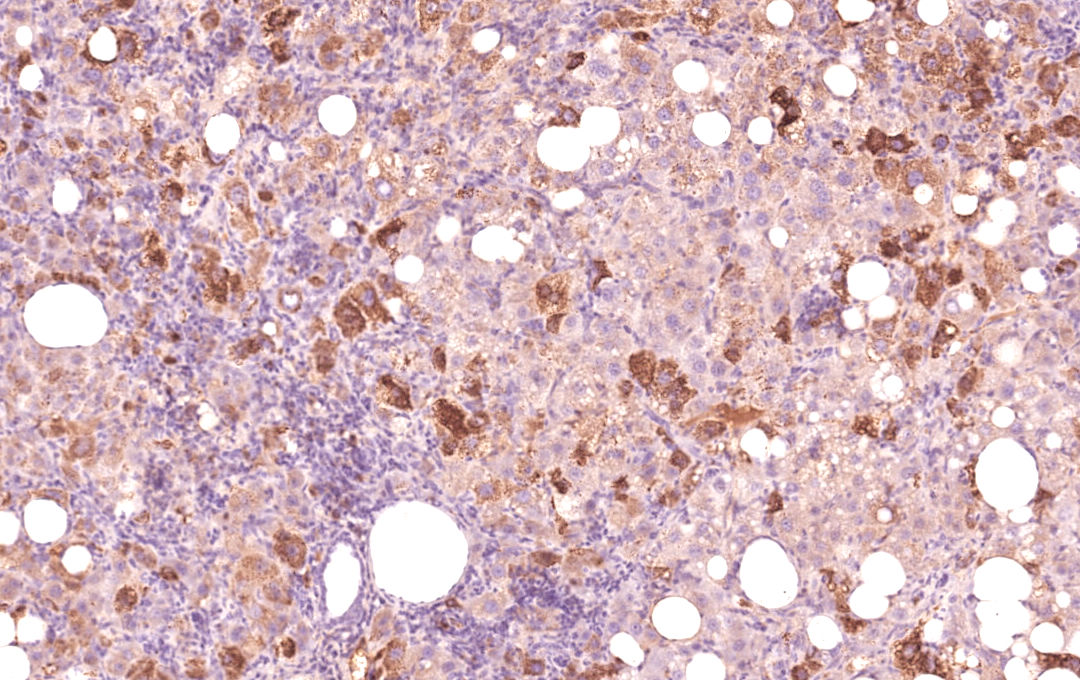

Supplement: Supplementary file 7 — Source Data Fig. 6 [file 44319_2024_71_MOESM7_ESM.zip › Figure 6/6F/IHC_Sirt6 LKO ob-HepPar1.jpg]

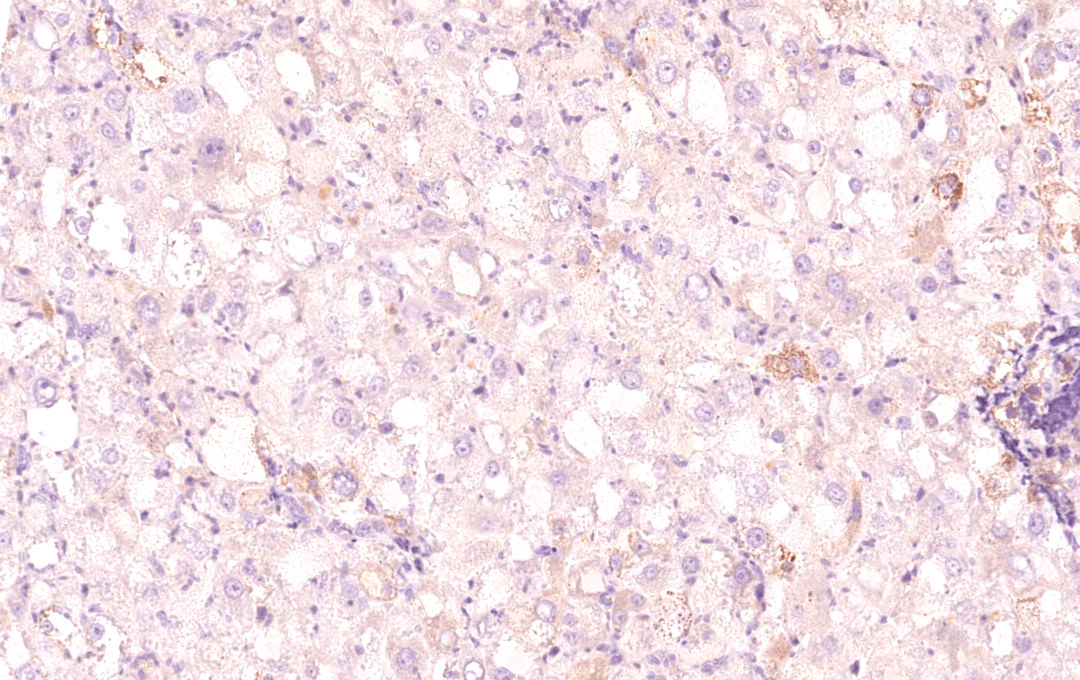

Supplement: Supplementary file 7 — Source Data Fig. 6 [file 44319_2024_71_MOESM7_ESM.zip › Figure 6/6F/IHC_Sirt6 Flox ob-HepPar1.jpg]

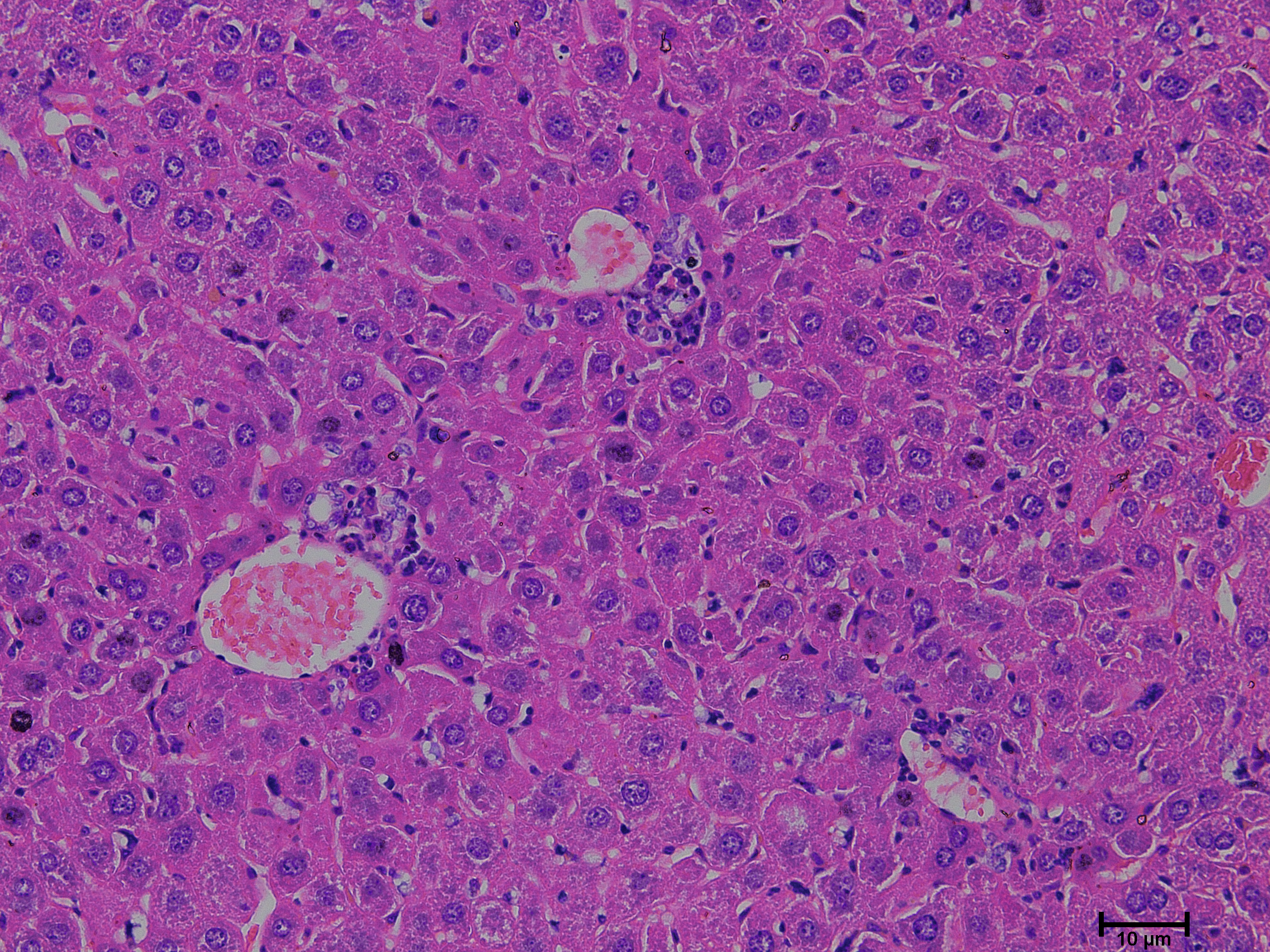

Supplement: Supplementary file 7 — Source Data Fig. 6 [file 44319_2024_71_MOESM7_ESM.zip › Figure 6/6A/HE Sirt6 Flox 2Y.tif]

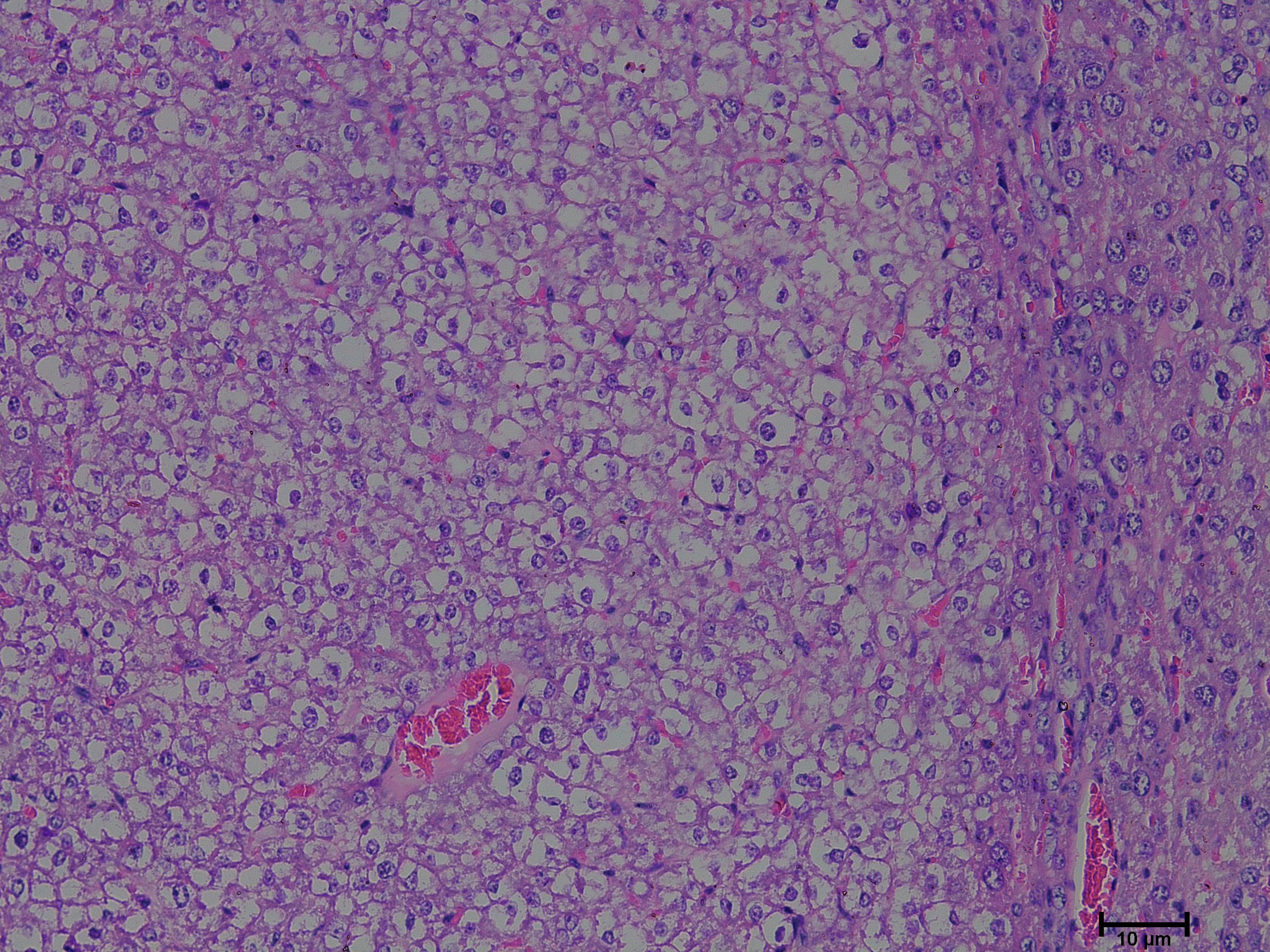

Supplement: Supplementary file 7 — Source Data Fig. 6 [file 44319_2024_71_MOESM7_ESM.zip › Figure 6/6A/HE Sirt6 LKO 2Y.tif]

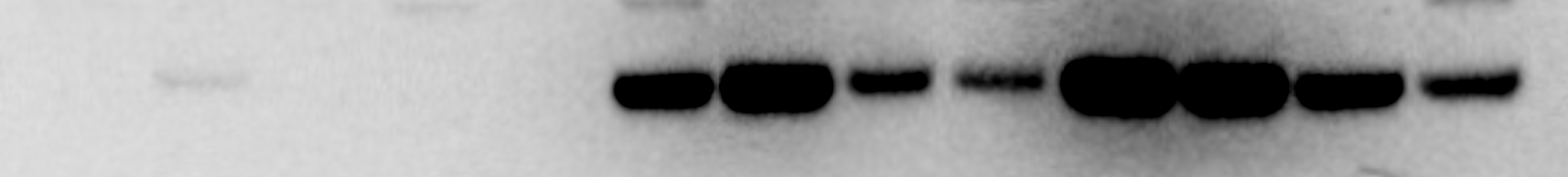

Supplement: Supplementary file 7 — Source Data Fig. 6 [file 44319_2024_71_MOESM7_ESM.zip › Figure 6/6G/Western Serpina12 ob.tif]

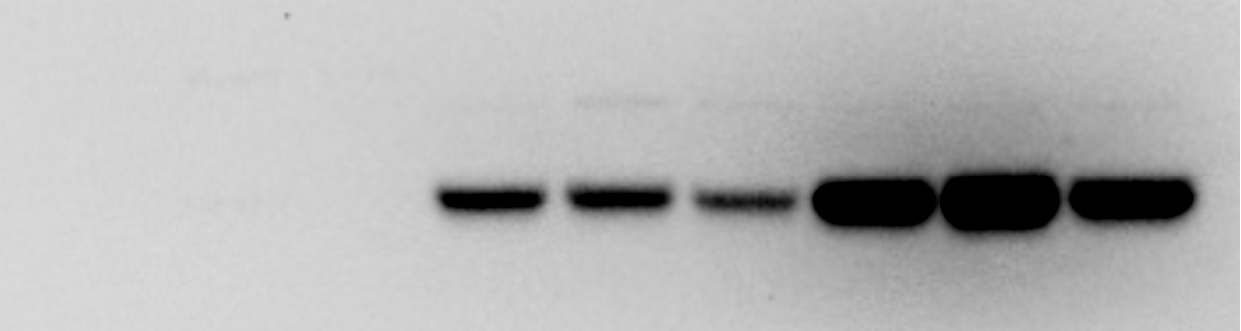

Supplement: Supplementary file 7 — Source Data Fig. 6 [file 44319_2024_71_MOESM7_ESM.zip › Figure 6/6G/Western Serpina12 den.tif]

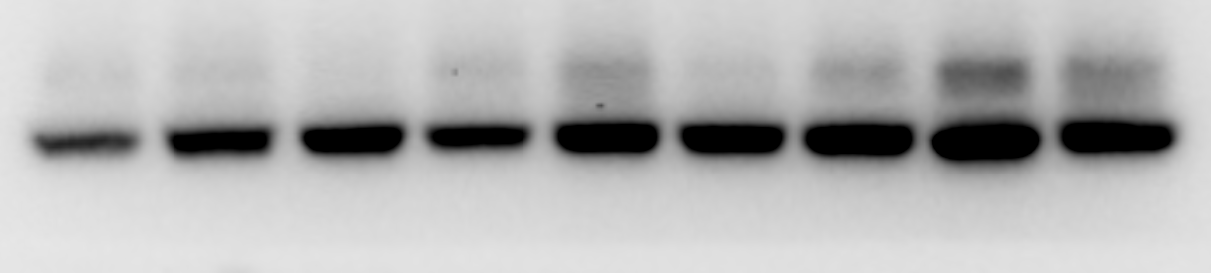

Supplement: Supplementary file 7 — Source Data Fig. 6 [file 44319_2024_71_MOESM7_ESM.zip › Figure 6/6G/Western actin den.tif]

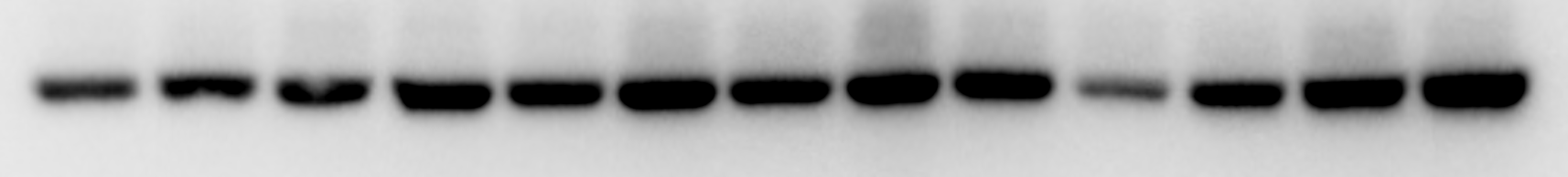

Supplement: Supplementary file 7 — Source Data Fig. 6 [file 44319_2024_71_MOESM7_ESM.zip › Figure 6/6G/Western actin ob.tif]

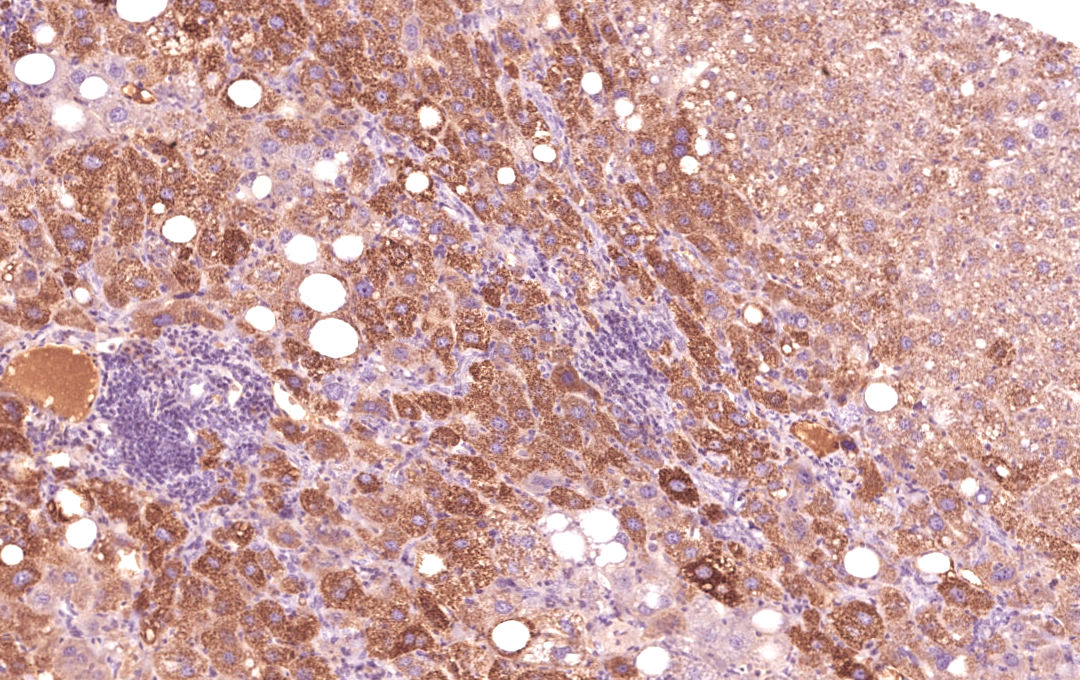

Supplement: Supplementary file 7 — Source Data Fig. 6 [file 44319_2024_71_MOESM7_ESM.zip › Figure 6/6B/IHC_Sirt6 LKO 2Y-HepPar1.jpg]

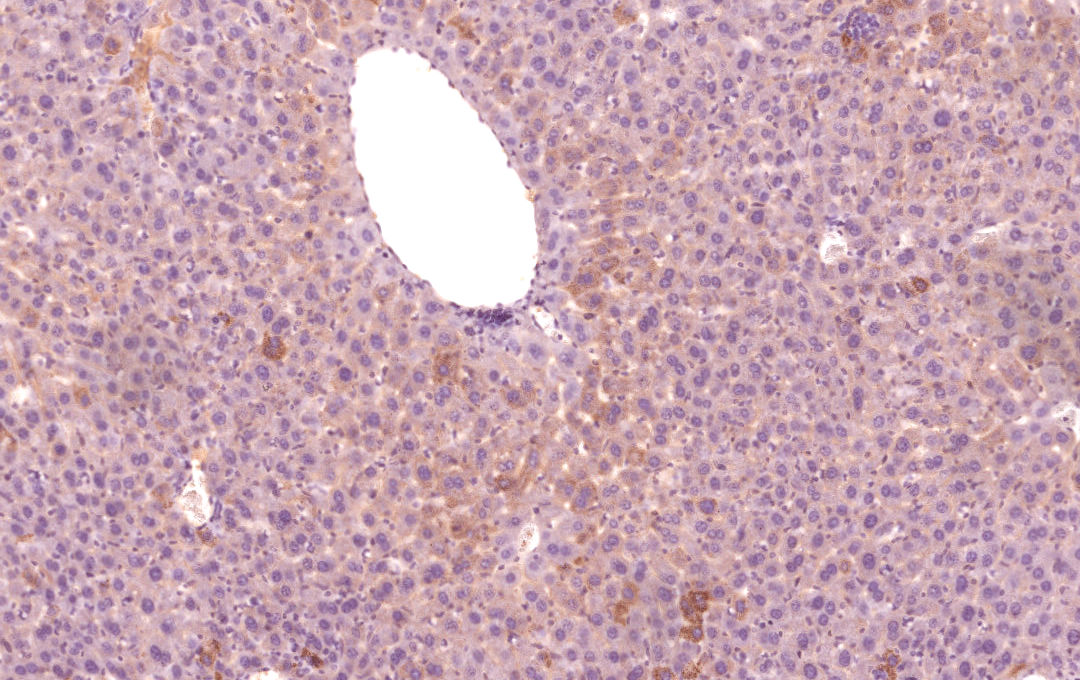

Supplement: Supplementary file 7 — Source Data Fig. 6 [file 44319_2024_71_MOESM7_ESM.zip › Figure 6/6B/IHC_Sirt6 Flox 2Y-HepPar1.jpg]

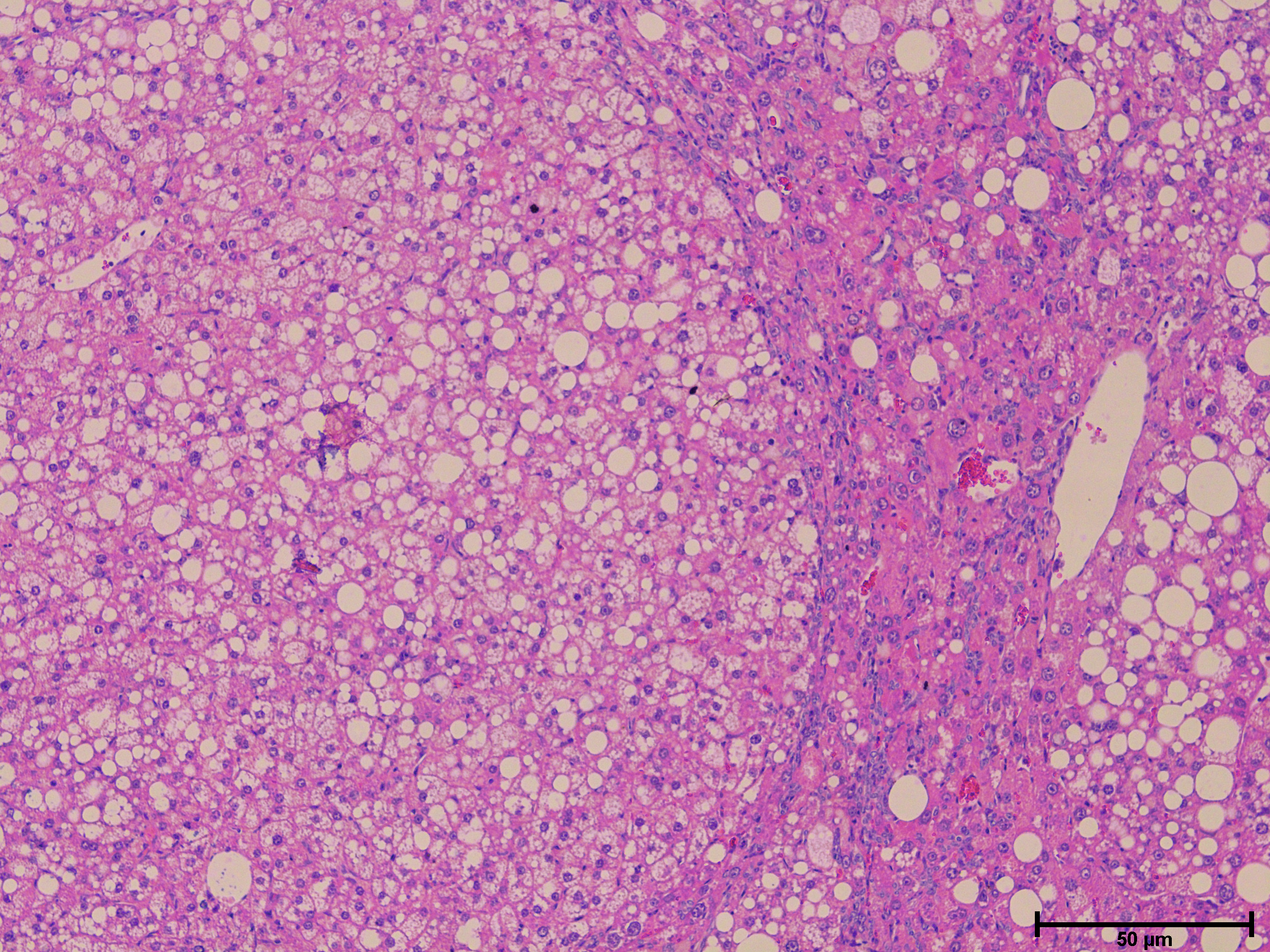

Supplement: Supplementary file 7 — Source Data Fig. 6 [file 44319_2024_71_MOESM7_ESM.zip › Figure 6/6E/HE Sirt6 LKO ob.tif]

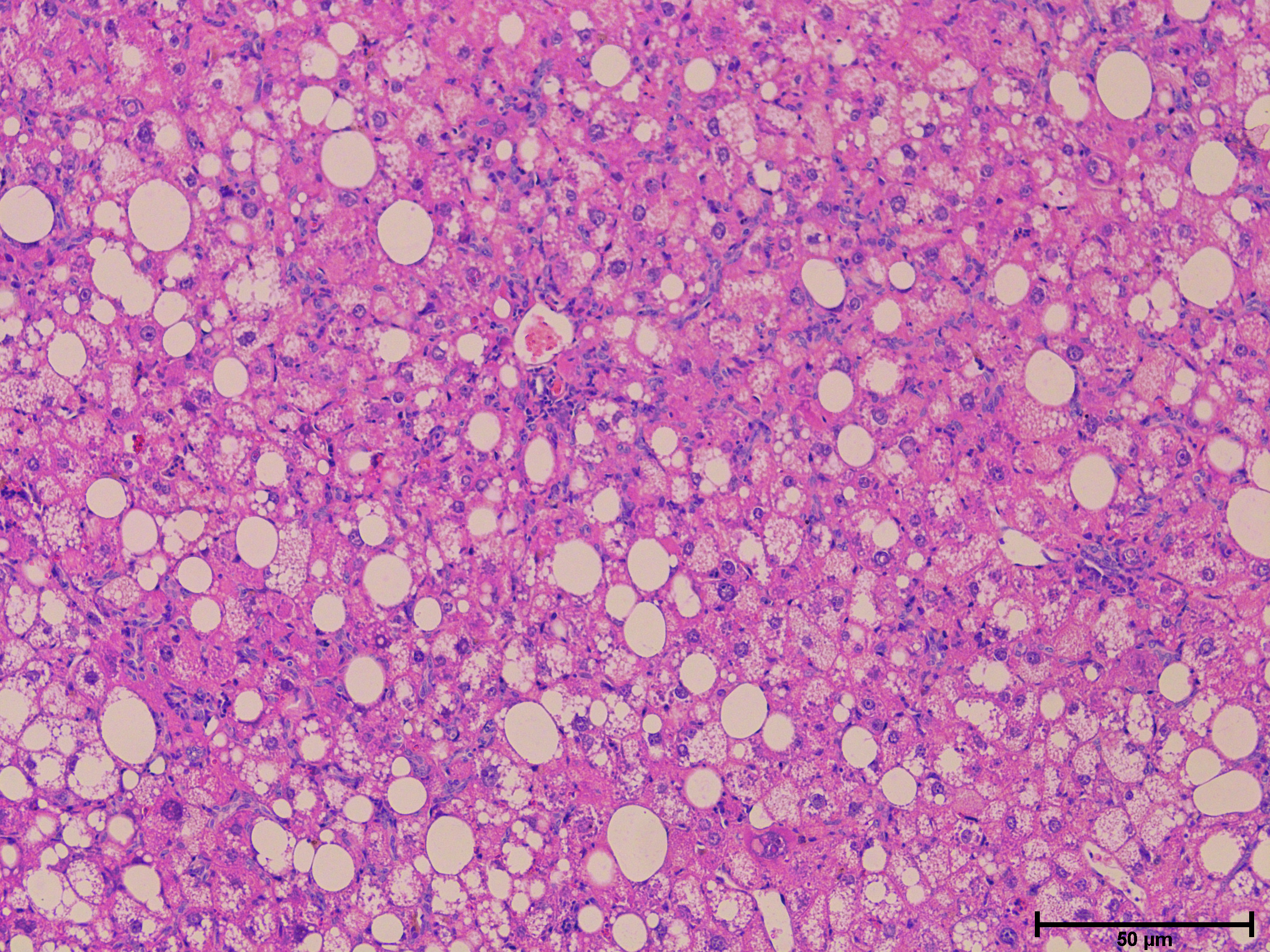

Supplement: Supplementary file 7 — Source Data Fig. 6 [file 44319_2024_71_MOESM7_ESM.zip › Figure 6/6E/HE Sirt6 Flox ob.tif]

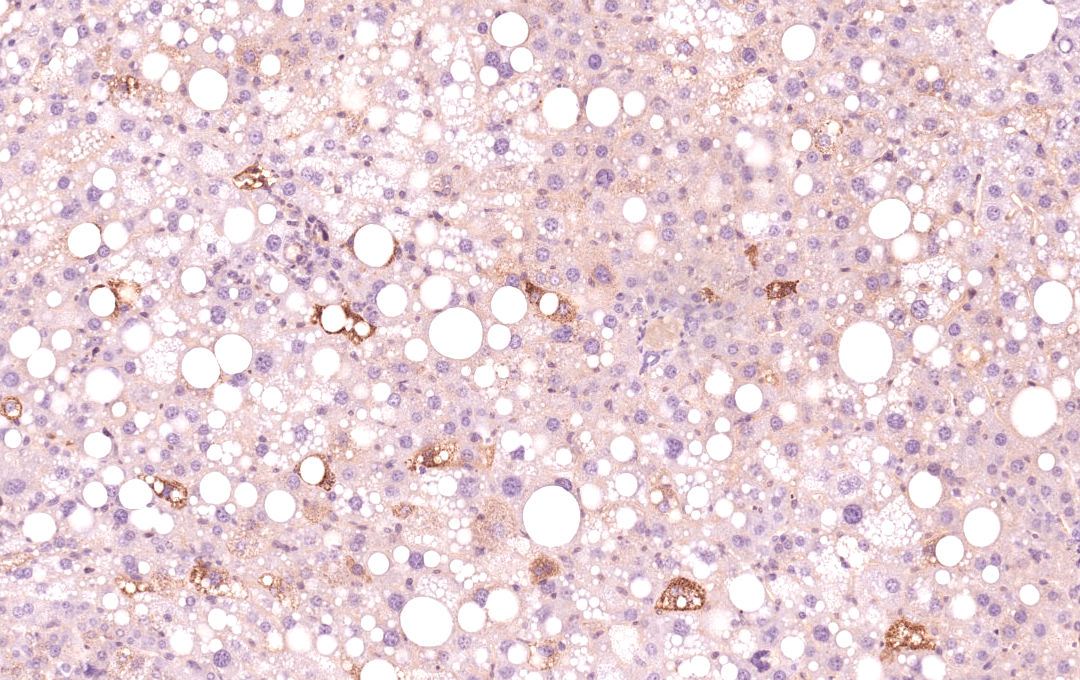

Supplement: Supplementary file 7 — Source Data Fig. 6 [file 44319_2024_71_MOESM7_ESM.zip › Figure 6/6D/IHC_Sirt6 Flox DEN-HepPar1.jpg]

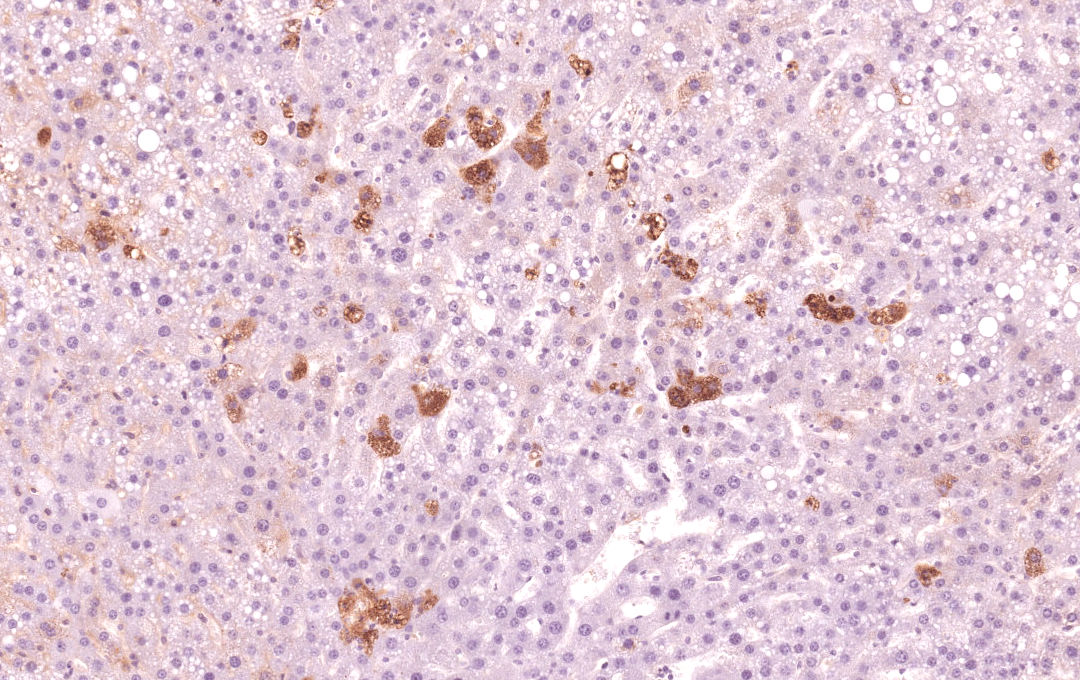

Supplement: Supplementary file 7 — Source Data Fig. 6 [file 44319_2024_71_MOESM7_ESM.zip › Figure 6/6D/IHC_Sirt6 LKO DEN-HepPar1.jpg]

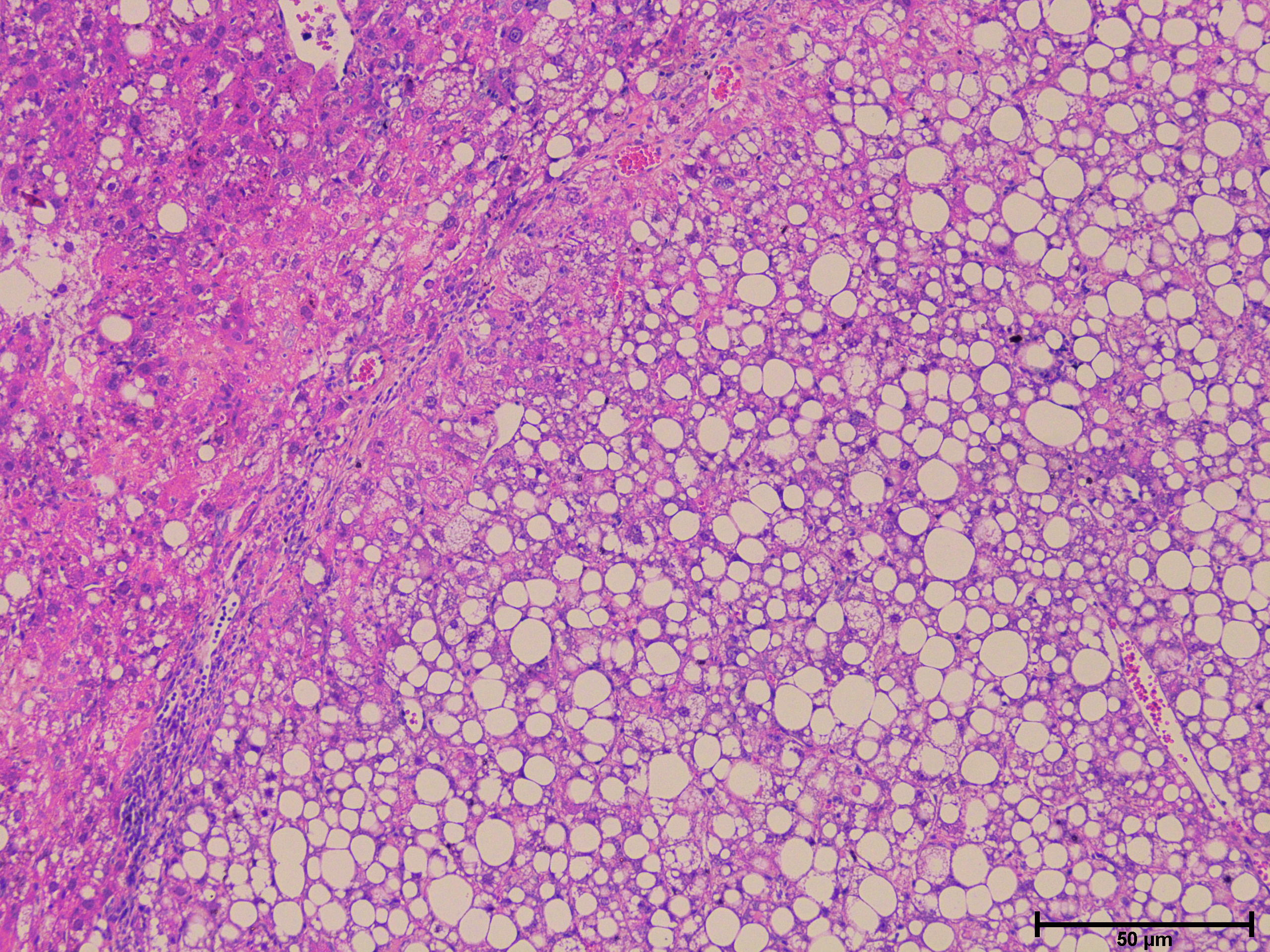

Supplement: Supplementary file 7 — Source Data Fig. 6 [file 44319_2024_71_MOESM7_ESM.zip › Figure 6/6C/HE Sirt6 LKO DEN.tif]

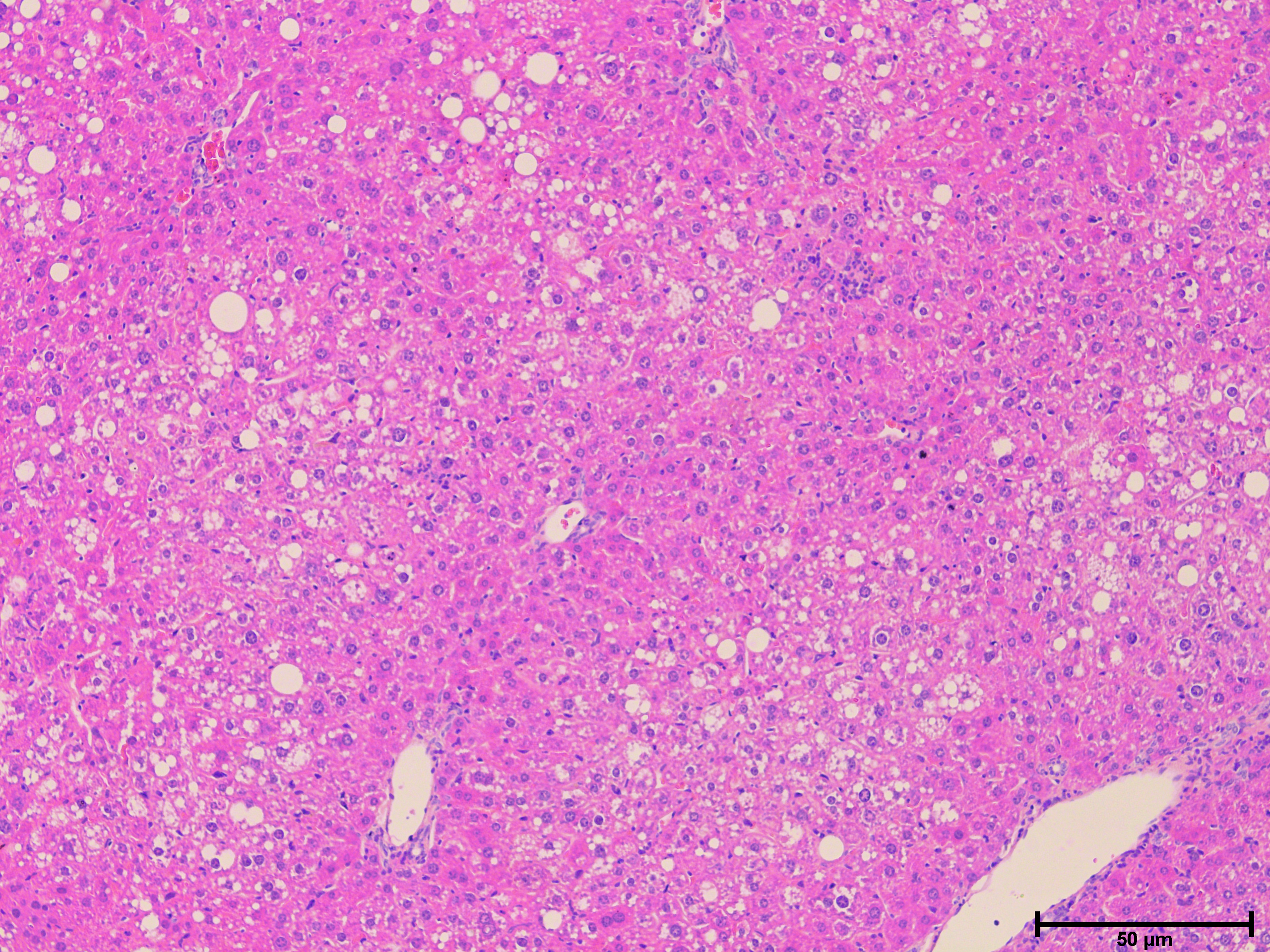

Supplement: Supplementary file 7 — Source Data Fig. 6 [file 44319_2024_71_MOESM7_ESM.zip › Figure 6/6C/HE Sirt6 Flox DEN.tif]
