# Supplementary material for: Sirt6 ablation in the liver causes fatty liver that increases cancer risk by upregulating Serpina12
Source: EMBO Rep. 2024 Feb 8;25(3):24. doi: 10.1038/s44319-024-00071-3 (PMC10933290; doi:10.1038/s44319-024-00071-3)
Supplement: Supplementary file 9 — Expanded View Figures [file 44319_2024_71_MOESM9_ESM.pdf]

Expanded View Figures

Figure EV1. Sirt6 ablation induces formation of fatty liver accompanied by alterations of a broad lipid-related gene expression and increased H3K9 and H3K56 acetylation.

(**A**) H&E staining and Oil red O staining of Sirt6 Floxed and Sirt6 LKO mice liver sections of 8 month old mice. Scale bars:100μm. (**B**) Expression levels of Sirt6 revealed by qPCR in Sirt6 Floxed or Sirt6 LKO mice. $n = 7$ BR; error bars = SEM. *t*-test. ****$p < 0.001$. (**C**) Expression levels of Sirt6 revealed by Western blot in Sirt6 Floxed or Sirt6 LKO mice. (**D**) RNA-seq showing downregulated different express genes enriched based on the biological process. $n = 3$ BR, *t*-test, p value < 0.05, Log_2 FC < −1. (**E**) Metagene analysis showing the bind pattern of H3K9ac or H3K56ac in Sirt6 Floxed or Sirt6 LKO mice liver. (**F**) Venn diagram showing that RNA-sequence upregulated genes and ChIP-H3K9Ac/H3K56Ac peaks. (**G**) Heatmap showed the 47 candidate genes' ranking list.

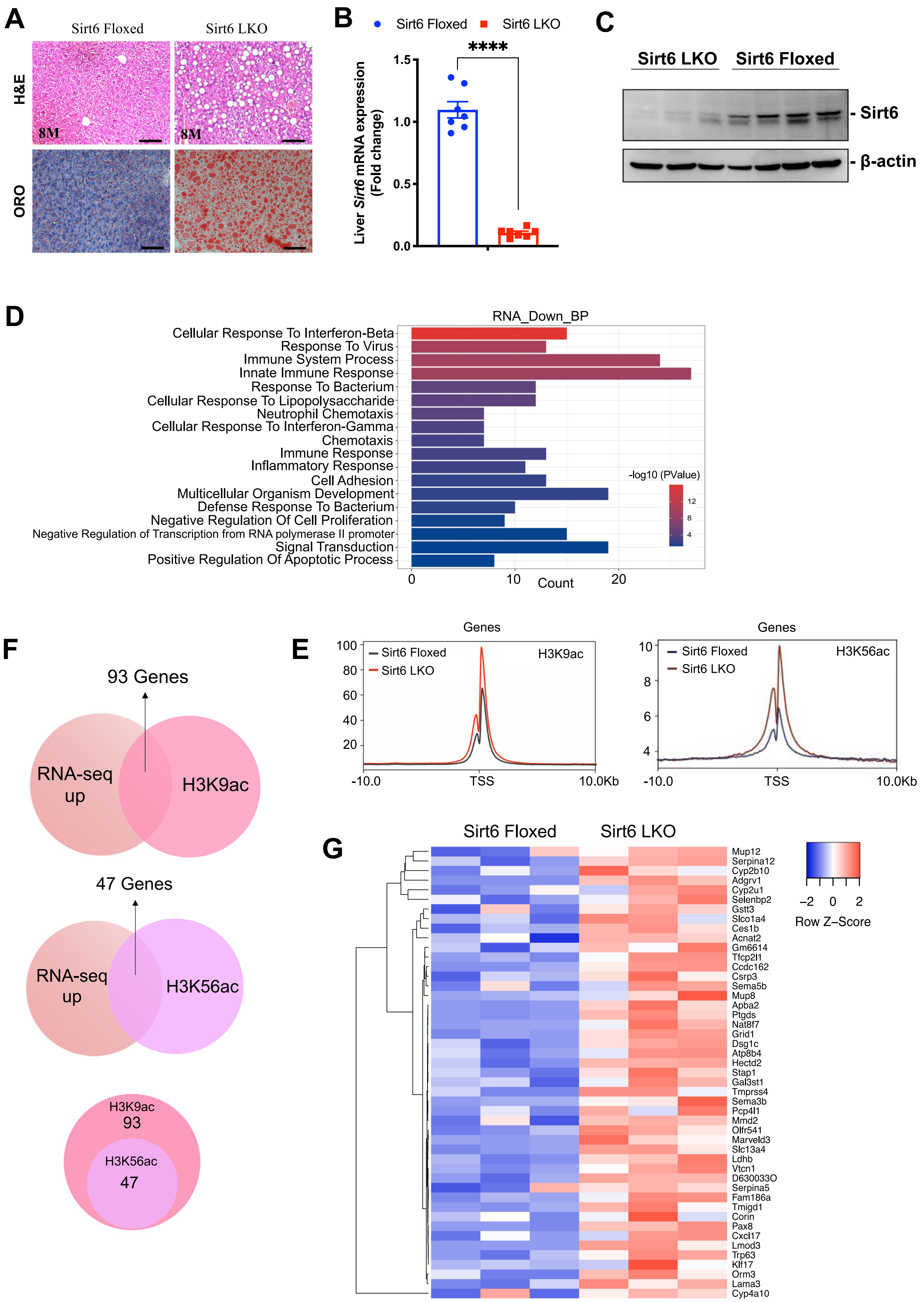
A
Sirt6 Floxed
Sirt6 LKO
H&E
ORO
8M
8M
B
Sirt6 Floxed
Sirt6 LKO
****
Liver Sirt6 mRNA expression (Fold change)
1.5
1.0
0.5
0.0
C
Sirt6 LKO
Sirt6 Floxed
- Sirt6
- β-actin
D
RNA_Down_BP
Cellular Response To Interferon-Beta
Response To Virus
Immune System Process
Innate Immune Response
Response To Bacterium
Cellular Response To Lipopolysaccharide
Neutrophil Chemotaxis
Cellular Response To Interferon-Gamma
Chemotaxis
Immune Response
Inflammatory Response
Cell Adhesion
Multicellular Organism Development
Defense Response To Bacterium
Negative Regulation Of Cell Proliferation
Negative Regulation of Transcription from RNA polymerase II promoter
Signal Transduction
Positive Regulation Of Apoptotic Process
-log10 (PValue)
12
8
4
0
10
Count
20
F
93 Genes
RNA-seq up
H3K9ac
47 Genes
RNA-seq up
H3K56ac
H3K9ac
93
H3K56ac
47
E
Genes
100
80
60
40
20
Sirt6 Floxed
Sirt6 LKO
H3K9ac
-10.0
TSS
10.0Kb
Genes
10
8
6
4
Sirt6 Floxed
Sirt6 LKO
H3K56ac
-10.0
TSS
10.0Kb
G
Sirt6 Floxed
Sirt6 LKO
Mup12
Serpina12
Cyp2b10
Adgrv1
Cyp2u1
Selenbp2
Gstt3
Slco1a4
Ces1b
Acnat2
Gm6614
Tfcp2l1
Ccdc162
Csrp3
Sema5b
Mup8
Apba2
Ptgds
Nat8f7
Grid1
Dsg1c
Atp8b4
Hectd2
Stap1
Gal3st1
Tmprss4
Sema3b
Pcp4l1
Mmd2
Olfr541
Marveld3
Slc13a4
Ldhb
Vtcn1
D630033O
Serpina5
Fam186a
Tmigd1
Corin
Pax8
Cxcl17
Lmod3
Trp63
Klf17
Orm3
Lama3
Cyp4a10
−2 0 2
Row Z−Score

 © The Author(s)

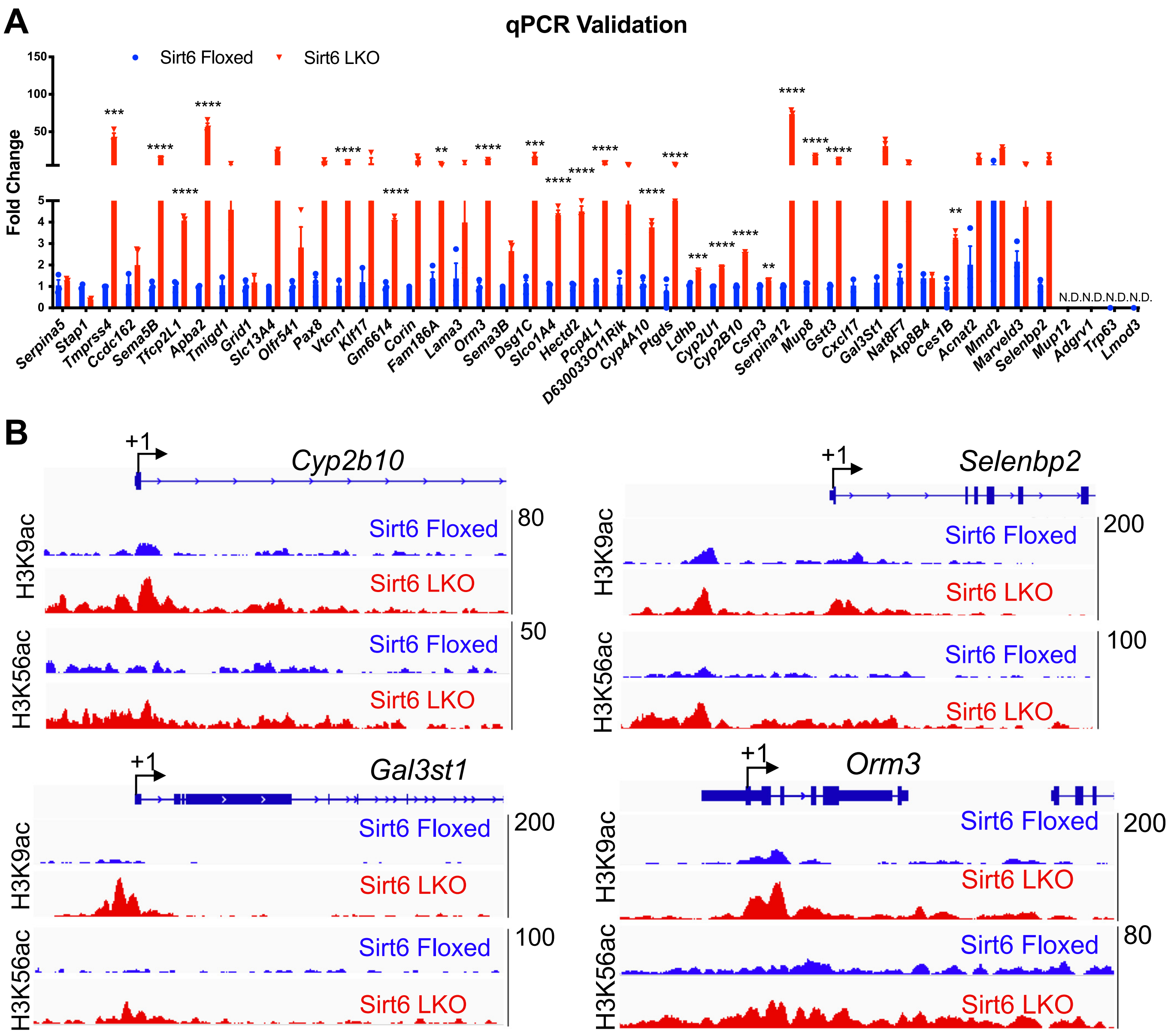

Figure EV2. Validation of candidates' expression and the levels of H3K9Ac and H3K56Ac around their promoters.

(**A**) qPCR validation of 47 candidate genes. Expression levels of Sirt6 revealed by qPCR in Sirt6 Floxed or Sirt6 LKO mice. $n = 3$ BR; error bars = SEM. Multiple *t*-test. **$p < 0.01$, ***$p < 0.005$, ****$p < 0.001$. (**B**) IGV browser of images of read coverage across the 4 candidate genes binding peaks in H3K9Ac and H3K56ac ChIP-seq from Sirt6 Floxed versus Sirt6 LKO mice liver.

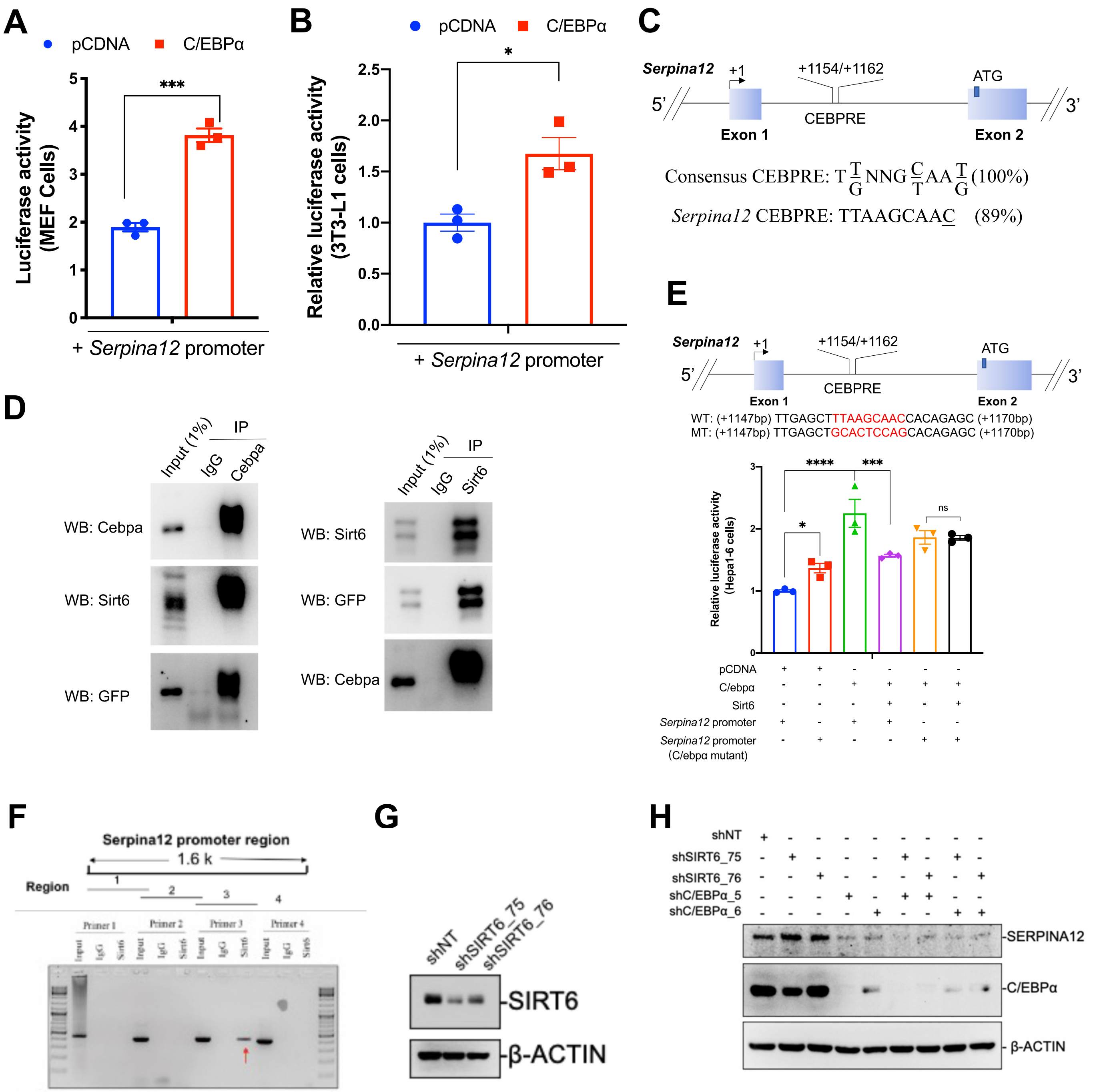

Figure EV3. Serpina12 is upregulated in the liver after sirt6 ablation through transcription factor CEBPα.

(**A**) Luciferase activity after transfected promoter *m-serpina12-0.75k* and CEBPα in MEF cells. $n = 3$ BR. (**B**) Luciferase activity after transfected promoter *m-serpina12-0.75k* and CEBPα in 3T3-L1 cells. $n = 3$ BR. (**C**) Consensus CEBPRE binding region on *Serpina12* gene region. (**D**) IP experiments on lysates from 293T/17 cells detected by antibodies of Sirt6 and C/ebpα. Sirt6-GFP and C/ebpα expression was induced 48 h prior to IP. (**E**) Ectopic expression of C/ebpα increased Serpina12 promoter activity, whereas mutation of C/ebpα-binding sites reduced the ienduction. Ectopic expression of Sirt6 represses the Serpina12 promoter activity but not the mutant C/ebpα-binding sites promoter activity. $n = 3$ BR. (**F**) SIRT6 ChIP to analysis SERPINA12 promoter binding region in HepG2 cells. (**G**) Western blot analysis shows the SIRT6 protein level in SIRT6 WT and knockdown groups in HepG2 cells. (**H**) Western blot analysis shows the SERPINA12 protein level with either knockdown SIRT6 or CEBPα in HepG2 cells. Data information: For (**A**),(**B**) and (**E**), error bars = SEM. *t*-test or two-way ANOVA. $*p < 0.05$, $***p < 0.005$, $****p < 0.001$, ns no significant difference.

 © The Author(s)

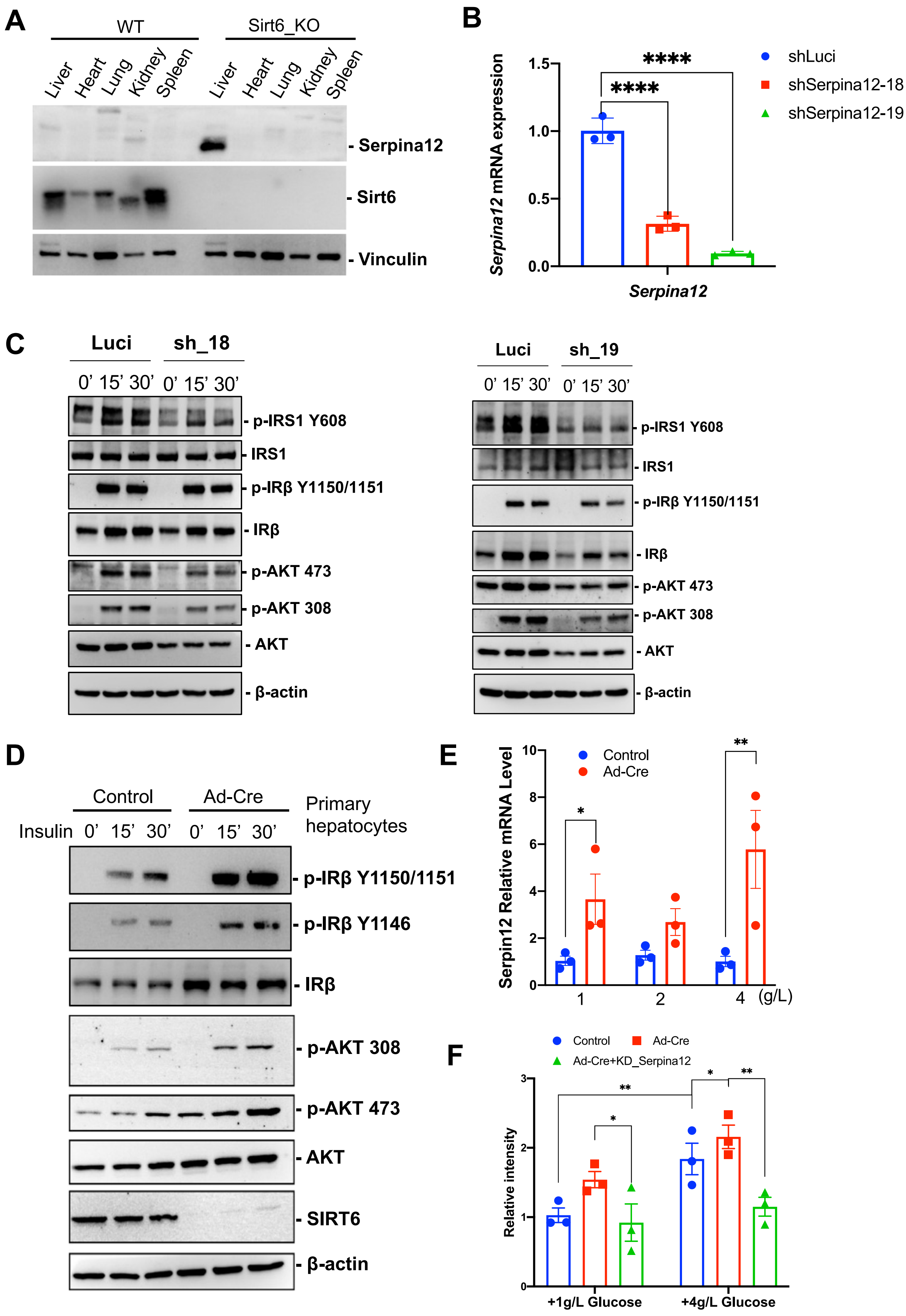
A
WT
Sirt6_KO
Liver
Heart
Lung
Kidney
Spleen
- Serpina12
- Sirt6
- Vinculin
B
shLuci
shSerpina12-18
shSerpina12-19
Serpina12 mRNA expression
****
****
Serpina12
C
Luci
sh_18
0' 15' 30' 0' 15' 30'
- p-IRS1 Y608
- IRS1
- p-IRβ Y1150/1151
- IRβ
- p-AKT 473
- p-AKT 308
- AKT
- β-actin
Luci
sh_19
D
Control
Ad-Cre
Insulin 0' 15' 30' 0' 15' 30'
Primary hepatocytes
- p-IRβ Y1150/1151
- p-IRβ Y1146
- IRβ
- p-AKT 308
- p-AKT 473
- AKT
- SIRT6
- β-actin
E
Control
Ad-Cre
Serpin12 Relative mRNA Level
(g/L)
F
Control
Ad-Cre
Ad-Cre+KD_Serpina12
Relative intensity
+1g/L Glucose
+4g/L Glucose

Figure EV4. SIRT6 affects insulin signaling by regulating the expression of Serpina12.

(**A**) Western blot shows the Serpina12 protein level in different tissue in SIRT6 WT or KO mice. (**B**) qPCR shows the knockdown efficiency of Serpina12 in primary hepatocytes. (**C**) Western blot analysis of insulin signaling following insulin treatment at 0 min, 15 min and 30 min by knockdown Luci control or two different Serpina12 shRNAs in primary hepatocytes. (**D**) Western blot analysis of insulin signaling following insulin treatment at 0 min, 15 min and 30 min in control or Ad-Cre in primary hepatocytes. (**E**) qPCR analysis of Serpina12 mRNA levels in different glucose concentration. (**F**) Relative intensity in control, knockout Sirt6 with or without knockdown Serpina12 primary hepatocytes. Data information: For (**B**),(**E**), and (**F**), $n = 3$ TR; error bars = SEM. Multiple *t*-test or two-way ANOVA. *$p < 0.05$, **$p < 0.01$, ****$p < 0.001$.

 © The Author(s)

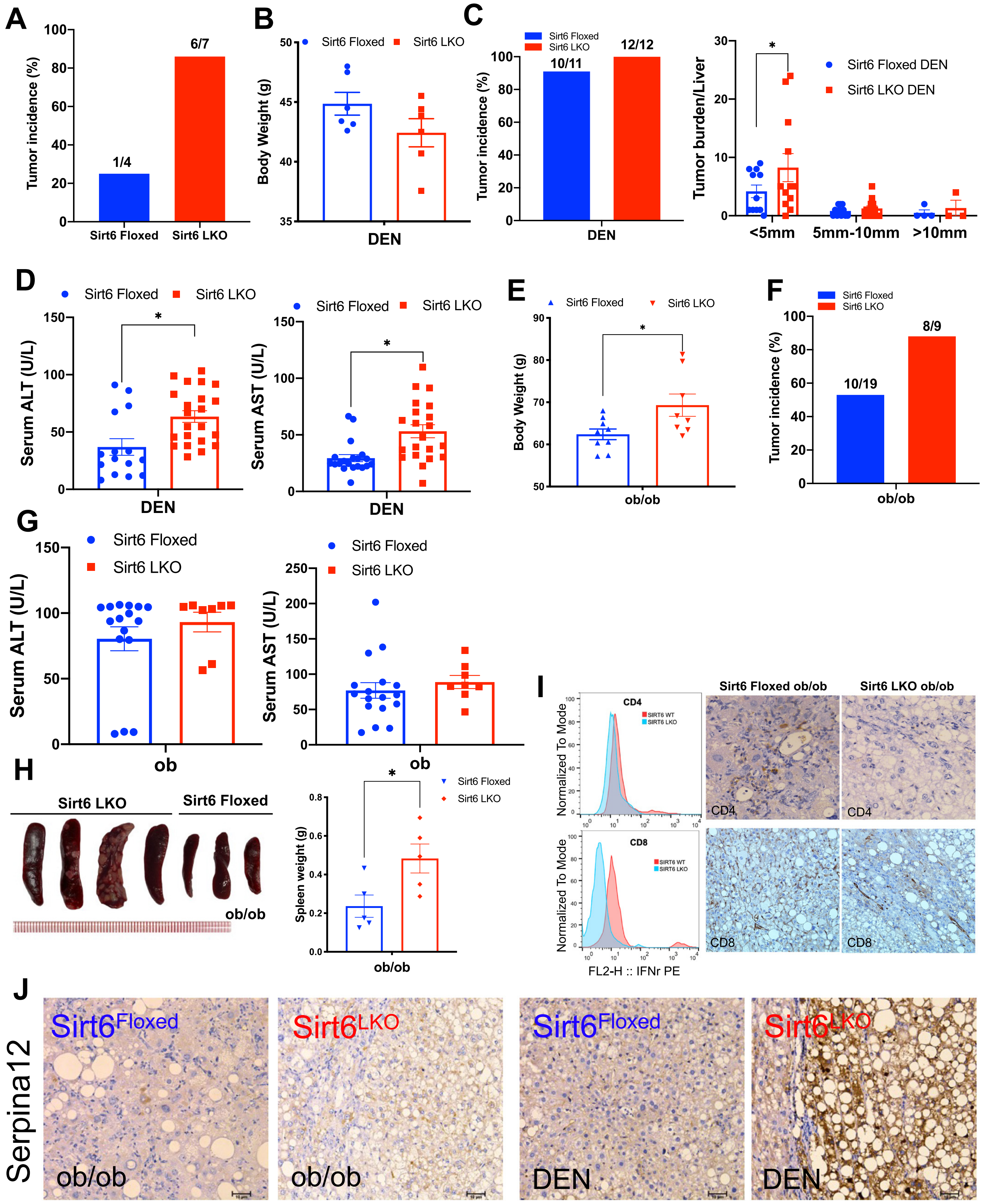
A
Tumor incidence (%)
1/4
6/7
Sirt6 Floxed
Sirt6 LKO
B
Sirt6 Floxed
Sirt6 LKO
Body Weight (g)
DEN
C
Sirt6 Floxed
Sirt6 LKO
10/11
12/12
Tumor incidence (%)
DEN
Tumor burden/Liver
Sirt6 Floxed DEN
Sirt6 LKO DEN
<5mm
5mm-10mm
>10mm
D
Sirt6 Floxed
Sirt6 LKO
Serum ALT (U/L)
Serum AST (U/L)
DEN
E
Sirt6 Floxed
Sirt6 LKO
Body Weight (g)
ob/ob
F
Sirt6 Floxed
Sirt6 LKO
Tumor incidence (%)
10/19
8/9
ob/ob
G
Sirt6 Floxed
Sirt6 LKO
Serum ALT (U/L)
Serum AST (U/L)
ob
H
Sirt6 LKO
Sirt6 Floxed
ob/ob
Spleen weight (g)
I
CD4
CD8
Normalized To Mode
FL2-H :: IFNr PE
Sirt6 Floxed ob/ob
Sirt6 LKO ob/ob
J
Serpina12
Sirt6Floxed
Sirt6LKO
ob/ob
DEN

Figure EV5. Sirt6 deficiency causes lipid-rich environment and less CD8$^+$ T cells infiltration in the liver thus accelerate tumor formation.

(**A**) Tumor incident (%) of Sirt6 Floxed or Sirt6 LKO mice in 2-year-old. (**B**) Body weight of Sirt6 Floxed and Sirt6 LKO with DEN injection mice. $n = 6$ BR. (**C**) Tumor incident in Sirt6 Floxed and Sirt6 LKO with DEN injection mice; Tumor burden per liver of Sirt6 Floxed and Sirt6 LKO with DEN injection mice. $n = 11$ or 12 BR. (**D**) Serum ALT level and AST level of Sirt6 Floxed and Sirt6 LKO with DEN injection mice. For ALT, $n = 15$ or 22 BR; For AST, $n = 20$ or 21 BR; (**E**) Body weight of Sirt6 Floxed and Sirt6 LKO with ob/ob mice. $n = 8$ or 9 BR. (**F**) Tumor incident in Sirt6 Floxed and Sirt6 LKO with ob/ob mice (6–9-month-old). (**G**) Serum ALT level and AST level of Sirt6 Floxed and Sirt6 LKO with ob/ob mice. For ALT, $n = 16$ or 8 BR; For AST, $n = 17$ or 8 BR; (**H**) Spleen morphology of Sirt6 Floxed and Sirt6 LKO with ob/ob mice at 7-month-old; Spleen weight of Sirt6 Floxed and Sirt6 LKO with ob/ob mice at 7-month-old. $n = 5$ BR. (**I**) The CD4$^+$ and CD8$^+$ T cells amount in the spleen of Sirt6 Floxed and Sirt6 LKO with ob/ob mice; IHC staining of CD4$^+$ and CD8$^+$ in Sirt6 Floxed and Sirt6 LKO with ob/ob mice. (**J**) IHC staining of Seprina12 in Sirt6 Floxed and Sirt6 LKO with ob/ob or DEN injection mice. Data information: For (**B–E**),(**G**), and (**H**), error bars = SEM. *t*-test. *$p < 0.05$.

 © The Author(s)
